# Supplementary material for: Inference of transmission dynamics and retrospective forecast of invasive meningococcal disease
Source: PLoS Comput Biol. 2023 Oct 27;19(10):e1011564. doi: 10.1371/journal.pcbi.1011564 (PMC10655980; doi:10.1371/journal.pcbi.1011564)
Supplement: S1 Text — We included 2 last sections with the Supplementary Tables and Figures, a description of these is listed below. Table A. Tables with Wilxonxon signed rank significant statistical tests for the ARIMA vs each individual process-based model. Table B. Tables with Wilxonxon signed rank significant statistical tests for the SARIMA vs each individual process-based model. Table C. Tables with Wilxonxon signed rank significant statistical tests for the MME vs process-based model 3. Fig A. Heatmap of R0 and carriage prevalence for varying values of the contact rate and likelihood of infection upon carriage. In Figs A1-A3 in s1 Text, we varied the recovery rate from 3 to 60 days, as indicated in the legend. Fig B. Inverse Wavelet Transform and detrended IMD incident cases. Fig C. C1. Posterior estimate of model 1 state variables from an EAKF. C2. Simulation of model 1 with time-varying parameters estimates from an EAKF. C3. Simulation of model 1 with point parameters estimates from an IF-EAKF. Fig D. D1. Posterior estimate of model 2 state variables from an EAKF. D2. Simulation of model 2 with time-varying parameters estimates from an EAKF. D3. Simulation of model 2 with point parameters estimates from an IF-EAKF. Fig E. E1. Posterior estimate of model 3 state variables from an EAKF. E2. Simulation of model 3 with time-varying parameters estimates from an EAKF. E3. Simulation of model 3 with point parameters estimates from an IF-EAKF. Fig F. Visualization of the forecast at a 6-month forecast horizon for the individual models. Fig G. G1. Time series of the WIS at 1-month forecast horizon for the 5 individual models. G2. Time series of the WIS at 3-month forecast horizon for the 5 individual models. G3. Time series of the WIS at 6-month forecast horizon for the 5 individual models. Fig H. Time series of the WIS at 6-month forecast horizon for the dynamical mechanistic models. Fig I. I1. Mean weighted interval score for the 5 individual models. I2. Boxplot of the WIS for the 5 in [file pcbi.1011564.s001.pdf]

# Supplementary Information: Inference of transmission dynamics and retrospective forecast of invasive meningococcal disease

Jaime Cascante-Vega, Marta Galanti, Katharina Schley, Sen Pei, Jeffrey Shaman

October 17, 2023

## Historical retrospective forecast

The process-based model was constructed for the simulation of invasive meningococcal disease (IMD) and designed for the assimilation of case count incident data. We first analyzed the disease-free equilibrium (DFE) of the core model and computed the basic reproductive number $R_0$ using the next generation matrix [1]. We also calculated the non-DFE to set informed priors on the contact rate $\beta$ and the likelihood of infection $\theta$ for the data assimilation process. The next sections describe $R_0$ computation as well as the rationale behind the prior ranges chosen.

### Time series analysis

In Figure 2 of the main text, we presented the Local Wavelet Power Spectrum (LWPS). We averaged the LWPS across the full study period and use the period that maximizes power (see the lower plot in Figure B) to fix either the seasonality of IMD transmission (model **ii**) or likelihood of infection (model **iii**). We plotted the inverse wavelet transform (IWT) and compared it with the detrended IMD incidence as shown in the upper plot of Figure B. In general, there is good agreement between the IWT and the detrended normalized IMD incidence, particularly prior to the completion of the vaccine regime in 2011.

### The process-based models

We employed a stochastic version of the disease transmission model in which individuals flowing between compartments are sampled from a Binomial distribution, i.e. $p = 1 - \exp(-\tau \cdot \Delta t)$, where $\tau$ is the rate of transition from compartment $X$ to compartment $Y$. We show the transitions between compartments (see Figure 1 in the main text) using the convention $x2y$ to represent the number of individuals flowing from compartment X to compartment Y. Lastly, $b$ represent births, assumed all susceptible, and $d_x$ represent deaths in each compartment $X$.

$$\lambda = \beta \frac{C + I}{N}$$

$$
\begin{aligned}
b =&\text{Binom}\left(N, 1-\exp\left(-\Delta t \cdot \mu\right)\right)\\
d_x =&\text{Binom}\left(X, 1-\exp\left(-\Delta t \cdot \mu\right)\right)\\
s2c =&\text{Binom}\left(S, 1-\exp\left(-\Delta t \cdot \lambda\right)\right)\\
c2i =&\text{Binom}\left(C, 1-\exp\left(-\Delta t \cdot \alpha_2\theta\right)\right)\\
c2s =&\text{Binom}\left(C, 1-\exp\left(-\Delta t \cdot \alpha_1(1-\theta)\right)\right)\\
i2s =&\text{Binom}\left(I, 1-\exp\left(-\Delta t \cdot \gamma\right)\right)\\
\end{aligned}
\tag{1}
$$

The state variables and incident cases (inc) are updated per the following equations:

$$
\begin{aligned}
\Delta S =&b - s2c + c2s + i2s - d_s\\
\Delta C =&s2c - c2i - c2s - d_c\\
\Delta I =&c2i - i2s - d_i\\
\text{inc} =&c2i
\end{aligned}
\tag{2}
$$

## Disease-free equilibrium and basic reproductive number

We fitted 3 process-based models and 1 statistical model (see Methods section in the main text). The mechanistic models were equal in form but varied in the way seasonality is imposed, i.e. either via the transmission rate $\beta$ (model 2), the likelihood of infection $\theta$ (model 3), or constant $\beta$ and $\theta$ (model 1). We analyzed the steady-state dynamics of the process-based models using the next generation matrix (NGM) to compute the basic reproductive number $\mathcal{R}_0$. The DFE is given by $[S^\star, C^\star, I^\star] = [N, 0, 0]$. We calculated the terms of the process model that contribute to increases in the number of carriers $C$ and Invasive Meningococcal disease cases $I$ shown in each row of matrix $F$ below. Similarly, we calculated the terms that reduce the number of infected individuals in each compartment shown in each row of matrix $V$. We then computed the partial derivatives of the state variables $I$ and $C$, shown in matrices $\mathcal{F}$ and $\mathcal{V}$.

$$
\mathcal{F} = \begin{bmatrix} \beta\frac{C+I+\omega}{N}S \\ 0 \end{bmatrix}, \quad \mathcal{V} = \begin{bmatrix} \alpha_2\theta C + \alpha_1(1-\theta)C \\ -\alpha_2\theta C + \gamma I \end{bmatrix}
\tag{3}
$$

$$
F = \begin{bmatrix} \beta & \beta \\ 0 & 0 \end{bmatrix}, \quad V = \begin{bmatrix} \alpha_2\theta + \alpha_1(1-\theta) & 0 \\ -\alpha_2\theta & -\gamma \end{bmatrix}
\tag{4}
$$

The next generation matrix (NGM) is computed as $NGM = FV^{-1}$ [1].

$$
NGM = \begin{bmatrix} \frac{\beta(-\alpha_2\theta+\gamma)}{\gamma(-\alpha_1\theta+\alpha_1+\alpha_2\theta)} & -\frac{\beta}{\gamma} \\ 0 & 0 \end{bmatrix}
\tag{5}
$$

$R_0$ is the largest eigenvalue of the NGM, i.e.

$$
R_0 = \frac{\beta}{\gamma}\frac{\gamma - \alpha_2\theta}{\alpha_1(1-\theta) + \alpha_2\theta}
\tag{6}
$$

## Non-DFE equilibrium

We computed the non-DFE that satisfies $dS/dt = 0$, $dI/dt = 0$ and $dC/dt = 0$. The system of equations that solve for this equilibrium is given below in Equation 7. We solved for the state variables as shown in Equations 8.

$$N = S + C + I$$
$$\lambda = \beta \frac{C + I}{N}$$

$$\begin{aligned} \mu N - \lambda S + \alpha_1(1-\theta)C + \gamma I - \mu S = 0 \\ \lambda S - \alpha_1(1-\theta)C - \alpha_2\theta C - \mu C = 0 \\ \alpha_2\theta C - \gamma I - \mu I = 0 \end{aligned} \tag{7}$$

$$\begin{aligned} \bar{S} =& \frac{N\left(-\alpha_1\gamma\theta + \alpha_1\gamma - \alpha_1\theta\mu + \alpha_1\mu + \alpha_2\gamma\theta + \alpha_2\theta\mu + \gamma\mu + \mu^2\right)}{\beta\left(\alpha_2\theta + \gamma + \mu\right)} \\ \bar{C} =& -\frac{N\left(\gamma + \mu\right)\left(-\alpha_1\gamma\theta + \alpha_1\gamma - \alpha_1\theta\mu + \alpha_1\mu - \alpha_2\beta\theta + \alpha_2\gamma\theta + \alpha_2\theta\mu - \beta\gamma - \beta\mu + \gamma\mu + \mu^2\right)}{\beta\left(\alpha_2\theta + \gamma + \mu\right)^2} \\ \bar{I} =& \frac{\alpha_2\theta\bar{C}}{\gamma + \mu} \end{aligned} \tag{8}$$

## Prior ranges

Our goal is to estimate the contact rate $\beta$, and the likelihood of infection $\theta$. The prior range of $\beta$ is informed by 2 empirical restrictions. First, we restricted $R_0 \approx 1$, and second that the prevalence of carriage between $\bar{C} = [1\%, 30\%]$. We solve Equations 6 and 7 for $\theta$ and $\beta$. Figures A1-A3 show the values of $R_0$ and $\bar{C}$ for different values of $\gamma$. We showed that considering the likelihood of infection $\theta \in [0, 1] \times 10^{-6}$, and $\beta \in [0.006, 0.01]$ satisfies the conditions required, that is $R_0 \in [0, 1.5]$ and $\bar{C} \in [0, 30\%]$. We chose 3 values for the time from carriage acquisition to disease, 1/60, 1/7 and 1/3, shown in Figures A1, A2 and A3, respectively, to study the sensitivity of $R_0$ and the prevalence $\bar{C}$, and shown that both quantities were not sensitive to this parameter.

## The Ensemble Adjustment Kalman Filter (EAKF)

The prior of the Markov process consists of 300 model simulations, which are collectively referred to as an ensemble. Given a current observation of incident IMD, $z_t$ at time $t$, we estimate the random error of observations assuming an observational error variance proportional to the incident IMD $i_t \sim \mathcal{N}(z_t, oev)$, as shown below in Eq. 9. Model simulations of the full ensemble provide a distribution of incident IMD, $\mathbf{y}_t$, $\mathbf{y}_t = [y_t^1, y_t^2, y_t^3, \ldots, y_t^{300}]$. The mean and variance of this distribution (the priors) can be calculated assuming normality, i.e. $y_t^i \sim \mathcal{N}(\mu_{prior}, \sigma^2_{prior})$. Using these moments, observations and the EAKF, we compute the posterior variance and mean as shown below [2].

$$\text{oev} = 1 + (p \cdot z_t)^2 \tag{9}$$

$$\sigma^2_{post} = \sigma^2_{prior} \frac{oev}{\sigma^2_{prior} + oev}$$
$$\mu_{post} = \sigma^2_{post} \left( \frac{\mu_{prior}}{\sigma^2_{prior}} + \frac{z_t}{oev} \right) \tag{10}$$

The Kalman gain $dy$ of each ensemble member $\mathbf{y}_t$ is given by

$$\mathbf{dy}_t = (\mu_{post} - \mathbf{y}_t) + \sqrt{\frac{oev}{oev + \sigma^2_{prior}}} (\mathbf{y}_t - \mu_{prior}) \tag{11}$$

The EAKF then uses the covariance between the parameters and the observations to compute the Kalman gain of the parameters $d\theta$ of each ensemble member for each set of parameters $\theta = [\theta_1, \theta_2, \ldots, \theta_{300}]$. Similarly, the Kalman gain of the state space $\boldsymbol{dx}$ is computed as the covariance of the state variables and the observations.

$$\mathbf{d}\theta = \frac{cov(\theta, \mathbf{y}_t)}{\sigma^2_{prior}} \times \mathbf{dy} \tag{12}$$

$$\mathbf{dx} = \frac{cov(\mathbf{x}, \mathbf{y}_t)}{\sigma^2_{prior}} \times \mathbf{dy} \tag{13}$$

Here $\theta_i$ is the tuple of ensemble members. The posterior observed state variable $\mathbf{y}_{post}$, unobserved state variables $\mathbf{x}_{post}$, and parameters $\theta_{post}$ are then given by

$$\mathbf{y_t}^{post} = \mathbf{y_t} + \boldsymbol{dy}$$
$$\mathbf{x_t}^{post} = \mathbf{x_t} + \boldsymbol{dx} \tag{14}$$
$$\boldsymbol{\theta}_{post} = \boldsymbol{\theta} + \boldsymbol{d\theta}$$

The pseudo-algorithm for the EAKF is shown below.

## Inference of transmission dynamics

We investigated the posterior inference of susceptibility, prevalence, and incidence using the EAKF as shown in the main text (Figure 3 A-C, for process model 3). We additionally performed an Iterated EAKF (IF-EAKF) to provide point estimates of parameters and study the simulated fit to the data with those parameters.

We compared the EAKF posterior inference of the state space of the 3 process-based models. Below we describe the process-based models and plot for each model the posterior inference using the EAKF, the simulations using the posterior parameter estimates from the EAKF, and simulations using the point estimates from the IF-EAKF. For all models and inference approaches we estimate the contact rate $\beta$ and the likelihood of infection $\theta$.

**Algorithm 1** Ensemble Adjustment Kalman Filter (EAKF)

**Input**

Parameter space across M ensemble members $\theta \in \mathcal{R}^{2\times M}$

Simulated incident at time $t$ for all ensemble members $\mathbf{y_t} \in \mathcal{Z}^M$

Simulated unobserved state variables at time $t$ for all ensemble members $\mathbf{x_t} \in \mathcal{Z}^{3\times M}$

Observed incident IMD at time $t$, $\mathbf{z_t} \in \mathcal{Z}^M$

Observed error variance $oev$.

Compute mean and variance of simulated colonization

$\mu_{\text{prior}} = \text{mean}(\mathbf{y_t})$

$\sigma^2_{\text{prior}} = \text{var}(\mathbf{y_t})$

Compute posterior mean and variance

$$\sigma^2_{\text{post}} = \sigma^2_{\text{prior}} \frac{\text{oev}}{\sigma^2_{\text{prior}} + \text{oev}}$$

$$\mu_{\text{post}} = \sigma^2_{\text{post}} \left( \frac{\mu_{\text{prior}}}{\sigma^2_{\text{prior}}} + \frac{z_t}{\text{oev}} \right)$$

Compute Kalman gain of observation and parameters space

$$\mathbf{dy} = (\mu_{\text{post}} - \mathbf{y_t}) + \sqrt{\frac{\text{oev}}{\text{oev} + \sigma^2_{\text{prior}}}} (\mathbf{y_t} - \mu_{\text{prior}})$$

$$\boldsymbol{dx} = \frac{cov(\boldsymbol{x}, \mathbf{y}_t)}{\sigma^2_{\text{prior}}} \times \mathbf{dy}$$

$$\boldsymbol{d\theta} = \frac{cov(\boldsymbol{\theta}, \mathbf{y}_t)}{\sigma^2_{\text{prior}}} \times \mathbf{dy}$$

Compute posterior observations and parameter space

$\mathbf{y_t^{post}} = \mathbf{y_t} + \mathbf{dy}$

$\mathbf{x_t^{post}} = \mathbf{x_t} + \mathbf{dx}$

$\boldsymbol{\theta}_{\mathbf{post}} = \boldsymbol{\theta_t} + \boldsymbol{d\theta}$

**Output:** Posterior state and parameters space

1. **Model 1:** Constant contact rate. Figure C1 shows the posterior estimate of the state variables using one iteration with the EAKF. Similar to the posterior of model 3 shown in the main text there is an increase in susceptibility from 2006 to 2011 with a jump in 2011 from around 91% to 96%. The susceptibility continues increasing until the end of the study period in agreement with the findings described in the main text. The simulation using the posterior parameter inference from the EAKF is shown in Figure C2. Consistent with the model structure the dynamics are not seasonal (See lower subplot in Figures C2 and C3), and there's also an increase in susceptibility starting from 87.5% in 2006 that increases up to 93% at the end of the study period in 2020 (upper left subplot); as a consequence, there is a decrease in prevalence (upper middle subplot). The compartment of infected individuals $I$ is the prevalence scaled by the likelihood of infection $\theta$, which follows the decreasing pattern (upper right subplot). The lower subplot shows the free simulation with estimated parameters. We find that observed IMD incidence generally falls between the 95% credible intervals (light salmon ribbon), and the mean simulated IMD incidence is in agreement with the decreasing trend seen in observations prior to 2011.

   The simulation using the point estimates from the IF-EAKF is shown in Figure C3. Here, again, susceptibility increases during the study period (upper left subplot), while prevalence decreases (upper middle subplot). The lower subplot of Figure C1 shows free simulations with the parameter estimates. Compared to the previous methods this simulation better captures the trend of the observed data with a constant decrease during the study period. However, the system is unable to represent seasonal variation, as this was not included in the model form.

2. **Model 2:** Here we imposed seasonality in the contact rate $\beta(t)$, using a sin function (Equation 15) $\beta_{amp}$ and $\beta_{base}$ are the amplitude and baseline of the sin function, which are estimated. The period $T$ is the time that maximizes the power of the LWPS averaged over the study period (Figure B).

$$\beta_(t) = \frac{\beta_{amp}}{2}\sin\left(2\pi\frac{t-1}{T}\right) + \left(\frac{\beta_{amp}}{2} + \beta_{base}\right) \tag{15}$$

   The posterior estimate of the state variables is shown in Figure D1, and the simulation using the posterior parameters estimates from the EAKF and the point estimates of the IF-EAKF are shown in Figures M and D3, respectively. We found an increase in the estimated susceptibility from the EAKF during the study period (upper left subplot in Figure D1). However, in contrast with the main text findings and the other 2 models, the susceptibility does not exhibit a jump in 2011 (upper left of Figure D1). We compared the simulated dynamics with the estimated parameters from the EAKF and the IF-EAKF and found that the fit to the IMD incident data is more in agreement when the IF-EAKF is used (lower subplot of Figures M and D3).

3. **Model 3:** Finally we imposed seasonality in the likelihood of infection $\theta(t)$. The expression is shown in Equation 16. $\theta_{amp}$ and $\theta_{base}$ are the amplitude and baseline of the sin function that are estimated. We use the period $T$ from the time series analyses as described before.

$$\theta(t) = \frac{\theta_{amp}}{2}\sin\left(2\pi\frac{t-1}{T}\right) + \left(\frac{\theta_{amp}}{2} + \theta_{base}\right) \tag{16}$$

   The posterior estimate of the state space is shown in Figure E1. We found an increase in the estimated susceptibility (upper left subplot) from the EAKF during the study period and the posterior estimates of the state space are in more agreement with observed IMD data than for the previous models shown in Figures C1 and D1. The simulation of the transmission dynamics from the parameter estimates of the EAKF and the IF-EAKF are shown in Figures M and D3, respectively. We found simulated IMD incidence to be in agreement with the decreasing

trend and seasonality observed in the data (lower subplots of each Figure) for both inference approaches. Again, the simulation with the parameter estimates is better when the IF-EAKF is used, rather than the EAKF (Figures E3 vs E2). The simulated dynamics are seasonal, as imposed by the model form, with periodicity given by the indicated period.

## Retrospective forecasting

Above, we showed that the model-inference system can simulate trajectories of historical outbreaks. We also produced retrospective forecasts with a 6-month forecast horizon. We used the EAKF to infer the state space and parameters until the forecast date and then integrated the model forward in time for 6 months. We repeated this process for all 3 model structures and the ARIMA model (see Methods section in the main text). Figure F shows some example 6-month forecasts. To enhance visual inspection of the forecasts we only show forecasts every 6 months. Generally, the ARIMA produced more uncertain forecasts when compared to the mechanistic models.

### The ARIMA model

We fit an autoregressive with integrating moving average (ARIMA) model implemented using the STATSMODEL package in PYTHON [3]. We analyzed partial auto-correlation (PACF) and auto-correlation (ACF) to choose the orders of the auto-regressive (p) and moving average terms (d), respectively. We used the order that satisfied a p-value greater than 0.05 and grid searched over possible combinations of (p, d). We fixed the order of integration to q=1.

### Individual model forecasts

We used the Mean Absolute Error (MAE) and the Weighted Interval Score (WIS), measures of the mean point fit and the probabilistic fit, respectively. The mathematical definitions of the scoring rules are shown below.

### Scoring rules

Let $F$ be a probabilistic forecast and $y$ the observation. Note that the scores are agnostic to time and therefore in the results presented in the following section we compute each score independently for the specified forecast horizon and then average over all forecasts.

1. **Median Absolute Error (MAE).**
   The MAE is computed as

   $$MAE = |\mathbb{E}\{F\} - y|$$

2. **Weighted Interval Score (WIS).**
   First, consider the interval score of a probabilistic forecast. The lower and upper quantiles of the probabilistic forecast $F$ given a confidence level $\alpha$ are denoted as $l_{F,\alpha}$ and $u_{F,\alpha}$ respectively.

   The interval score with confidence $\alpha$, $IS_\alpha(F, y)$, is computed using Equation 17. The indicator function is defined as $\mathbf{1}(y < l_{F,\alpha)}) = 1 \iff y < l_{F,\alpha)} \quad = 0, \text{otherwise}$.

   $$IS_\alpha(F, y) = (u_{F,\alpha} - l_{F,\alpha}) + \frac{2}{\alpha}(l_{F,\alpha} - y)\mathbf{1}(y < l_{F,\alpha)}) + \frac{2}{\alpha}(y - u_{F,\alpha})\mathbf{1}(y > u_{F,\alpha}) \qquad (17)$$

   The weighted interval score is computed as a weighted average over $K$ quantiles each one with confidence $\alpha_k$ [4].

$$WIS_\alpha(F,y) = \frac{1}{K+0.5}\left(\frac{1}{2}|y - \text{median}(F)| + \sum_{k=1}^{K} \alpha_k \cdot IS_{\alpha_k}(F,y)\right)$$

We report the historical performance for 6 different forecast horizons from 1 to 6 months. Figures G1-G3 show the performance of each model in each forecast horizon; the upper plot shows the MAE and the lower plot shows the WIS. The point score, the MAE, shows that the mechanistic models perform similarly over time, and in some time windows, the ARIMA performs better. We also investigated the probabilistic performance and found that the ARIMA is highly penalized, a consequence of its broad, uncertain forecast distribution (Figure F). Among the mechanistic models, we found that model 3 performed the best across the entire study period, although for some time windows model 1 performed better. All models performed poorly when predicting the unusual peaks in 2008 and 2019.

## Multi-model Ensemble (MME): Forecast aggregation.

We used the Expectation Maximization (EM) algorithm for computing weights to average the quantiles of the individual models [5]. The EM algorithm goal was used to determine a set of weights $w_i \quad \forall i \in [1, \text{number of models}]$ with the constraint $\sum_i w_i = 1$. The weights are computed iteratively using the Bayes rule and the likelihood of observing the data given the model $i$ $P(z|\text{model}_i)$. The weights of each iteration are computed by normalizing $P(\text{model}_i|\text{data})$ by the number of models. We use the WIS for defining this likelihood in two different fashions as indicated in the main text: using all past model WIS and using only the past $K$ months WIS. We normalize the WIS, so it sums up to 1.

$$P(\text{model}_i|\text{data}) = \sum_k \frac{w_i \cdot P(z|\text{model}_i)}{P(\text{data})} \tag{18}$$

### Individual model and MME performance

We compared the WIS of the MME and the individual models. Additionally, we constructed an equally weighted ensemble. Figure I1 and I2 show the mean WIS and the distribution of WIS, respectively. We evaluated performance during 3 time periods: the entire study period (upper subplot), before the completion of the vaccine regime, i.e. until 2011 (middle subplot), and after 2011 (lower subplot). We found that both the mean and the distribution of the WIS were highest for the ARIMA model indicating a poor performance. Similarly, we showed that both the mean and distribution of the WIS are better for model 3.

We used the Wilcoxon signed-rank test to assess statistically significant differences in the distribution of WIS between the ARIMA and each mechanistic model. We found that the mechanistic models performed better, and this finding was consistent across forecast horizons (Table A).

Figures J show the WIS using different lengths of past performance from 2 to 6 months. The results are similar indicating little sensitivity to the number of past months considered for MME; there is slightly better performance when using the 5 previous months.

Figures L1 and L2 show the weights assigned to each model (x-axis) during the study period for the MME constructed using 2 and 6 months past performance. We found the weights between the MME are similar across the study period in agreement with our previous findings and suggesting that the past months considered do not change the MME.

We also examined the weights when all past performance was considered (Figure L3). Here the model consistently principally weighted models 1 and 3.

# Tables

| Evaluation horizon | ARIMA vs Model 1 | ARIMA vs Model 2 | ARIMA vs Model 3 |
|---|---|---|---|
| 1 month | 1.29e-21 | 3.73e-18 | 1.04e-21 |
| 2 month | 2.88e-25 | 5.20e-24 | 2.44e-26 |
| 3 month | 2.32e-28 | 5.32e-27 | 1.04e-29 |
| 4 month | 5.91e-29 | 5.06e-29 | 5.93e-31 |
| 5 month | 3.89e-29 | 3.09e-30 | 1.37e-31 |
| 6 month | 4.98e-29 | 2.05e-30 | 2.23e-31 |

Table A: p-values of the Wilcoxon signed rank test of the WIS distribution during the study period. The forecast horizon is indicated in the rows, and each column shows the pair test between the ARIMA and each individual mechanistic model.

| Evaluation horizon | SARIMA vs Model 1 | SARIMA vs Model 2 | SARIMA vs Model 3 |
|---|---|---|---|
| 1 month | 1.07e-15 | 4.53e-13 | 1.16e-17 |
| 2 month | 7.18e-17 | 3.45e-17 | 2.86e-20 |
| 3 month | 1.94e-18 | 1.18e-19 | 2.24e-22 |
| 4 month | 5.76e-20 | 9.77e-22 | 6.91e-25 |
| 5 month | 1.51e-21 | 1.56e-23 | 2.14e-26 |
| 6 month | 1.32e-22 | 3.56e-24 | 2.11e-28 |

Table B: p-values of the Wilcoxon signed rank test of the WIS distribution during the study period. The forecast horizon is indicated in the rows, and each column shows the pair test between the SARIMA and each individual mechanistic model.

| | 1 m | 2 m | 3 m | 4 m | 5 m | 6 m |
|---|---|---|---|---|---|---|
| Ens. (2 Months) vs Ens. (All past) | 7.54e-01 | 8.58e-02 | 6.65e-02 | 2.32e-02 | 9.06e-04 | 1.58e-03 |
| Ens. (2 Months) vs Ens. (Eq. Weights) | 1.07e-08 | 4.87e-11 | 7.93e-13 | 1.31e-16 | 8.09e-17 | 1.33e-17 |
| Ens. (All past) vs Ens. (Eq. Weights) | 3.37e-12 | 3.21e-17 | 1.82e-20 | 4.89e-23 | 1.70e-24 | 1.39e-25 |
| Model 3 vs Ens. (2 Months) | 4.07e-01 | 8.22e-02 | 6.09e-02 | 2.13e-02 | 1.36e-03 | 7.28e-03 |
| Model 3 vs Ens. (All past) | 8.01e-01 | 2.43e-01 | 2.02e-01 | 1.14e-01 | 1.07e-01 | 5.60e-01 |
| Model 3 vs Ens. (Eq. Weights) | 6.31e-13 | 4.21e-18 | 4.68e-21 | 4.46e-24 | 2.13e-25 | 9.08e-26 |

Table C: p-values of the Wilcoxon signed rank test of the WIS distribution during the study period. The forecast horizon is indicated in the columns, and each row shows the pair of models being compared

# Figures

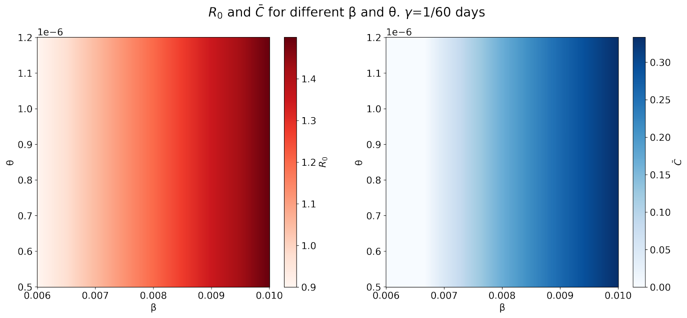


Fig A1: **Basic reproductive number $R_0$ and carriage prevalence (C)**. Heatmaps varying the likelihood of infection $\theta$ and the contact rate $\beta$. The left subplot shows the Basic reproductive Number $R_0$ and the right the prevalence (steady state of carriers). The value of the 'recovery rate' after infection is $\gamma = 1/60$ days.

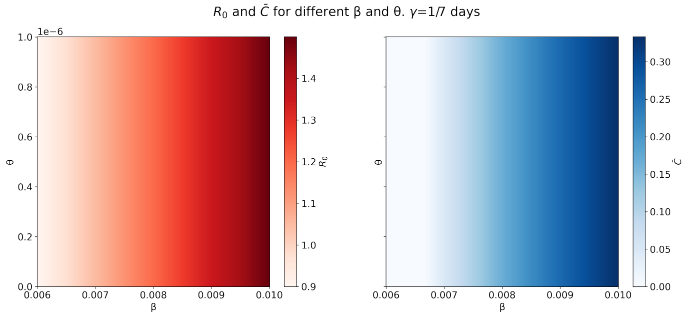


Fig A2: **Basic reproductive number $R_0$ and carriage prevalence (C)**. Heatmaps varying the likelihood of infection $\theta$ and the contact rate $\beta$. The left subplot shows the Basic reproductive Number $R_0$ and the right the prevalence (steady state of carriers). The value of the 'recovery rate' after infection is $\gamma = 1/7$ days.

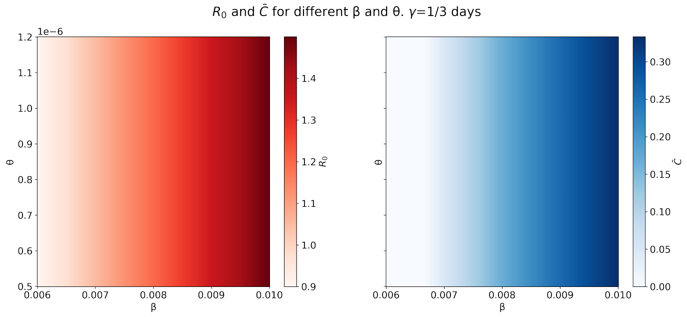


Fig A3: **Basic reproductive number $R_0$ and carriage prevalence (C)**. Heatmaps varying the likelihood of infection $\theta$ and the contact rate $\beta$. The left subplot shows the Basic reproductive Number $R_0$ and the right the prevalence (steady state of carriers). The value of the 'recovery rate' after infection is $\gamma = 1/3$ days.

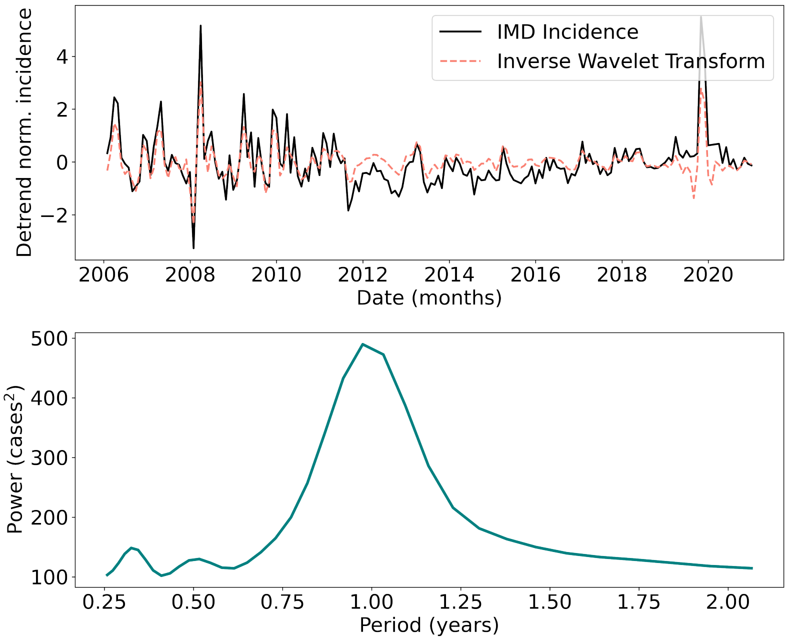


Fig B: Upper plot shows the IMD incident cases as a continuous black line and the inverse wavelet transform (IWT). The lower plot shows the average power spectrum across the study period.

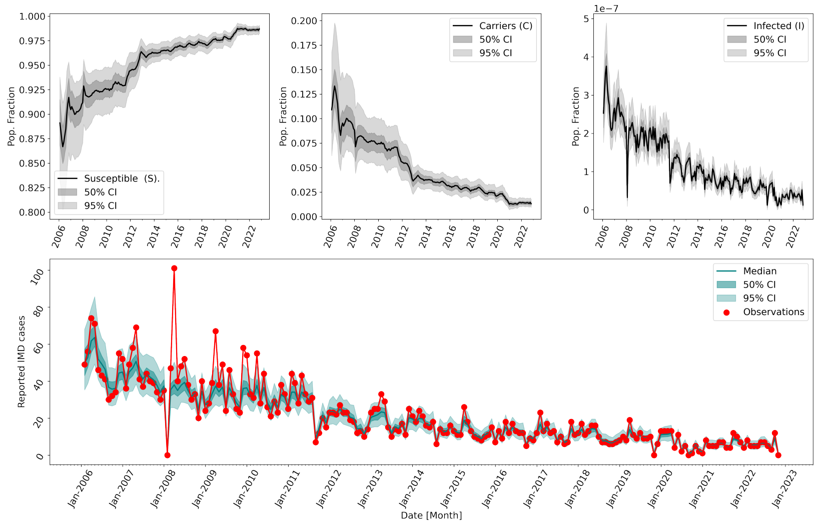


Fig C1: **Model 1. Constant contact rate**. Upper plots show the posterior estimates of the state variables of an EAKF pass. From left to right we plot Susceptibility (S), Prevalence (C), and IMD (I); the lower plot shows incident IMD and reported data as red dots.

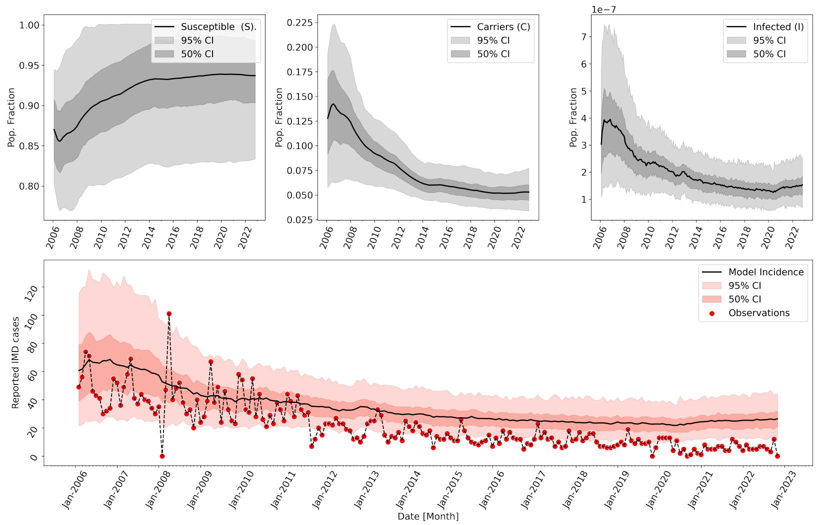


Fig C2: **Model 1. Constant contact rate. Posterior EAKF state variables**. Upper plots show the posterior estimates of the state variables of an EAKF pass. From left to right we plot Susceptibility (S), Prevalence (C), and IMD (I). Simulation is performed using the posterior parameter estimates from a single EAKF pass.

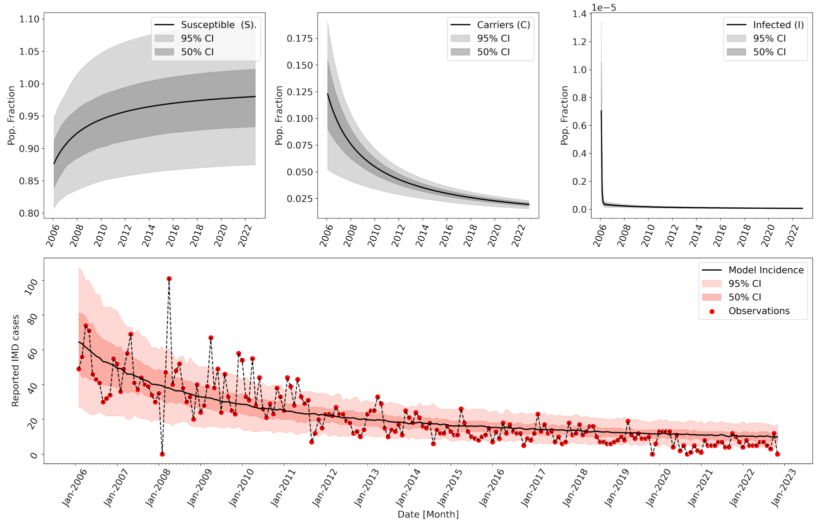


Fig C3: **Model 1. Constant contact rate. IF-EAKF MLE simulation** $\theta$. The upper plots show the time evolution of the state variables from left to right: Susceptibility (S), Prevalence (C), and IMD (I); the lower plot shows incident IMD and reported data as red dots. Simulation is performed using the MLE of the IF-EAKF.

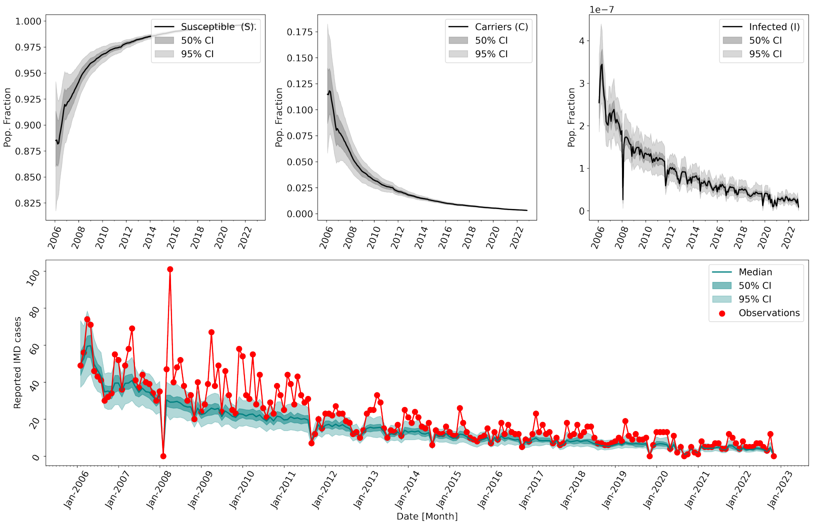


Fig D1: **Model 2. Seasonality in the contact rate. Posterior EAKF state variables**. Upper plots show the posterior estimates of the state variables of an EAKF pass. From left to right we plot Susceptibility (S), Prevalence (C), and IMD (I); the lower plot shows incident IMD and reported data as red dots.

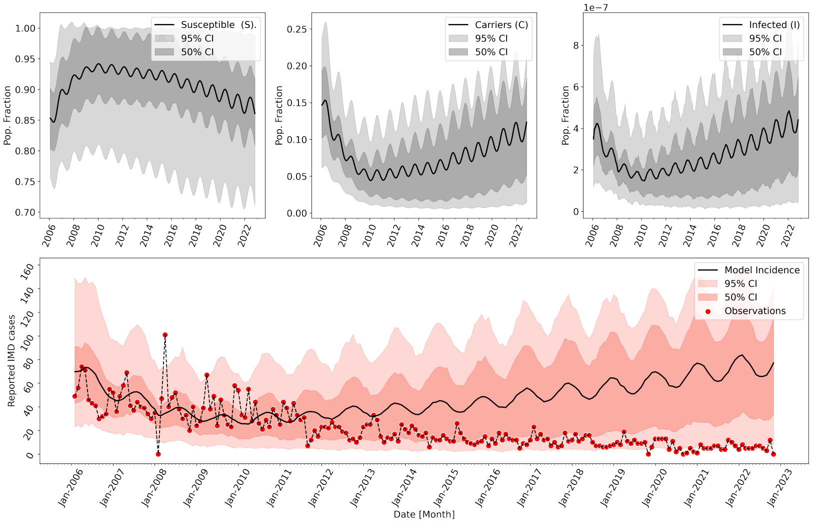


Fig D2: **Model 2. Seasonality in contact rate. EAKF simulation with posterior parameters.** $\theta$. The upper plots show the time evolution of the state variables from left to right: Susceptibility (S), Prevalence (C), and IMD (I). Simulation is performed using the posterior parameter estimates from a single EAKF pass.

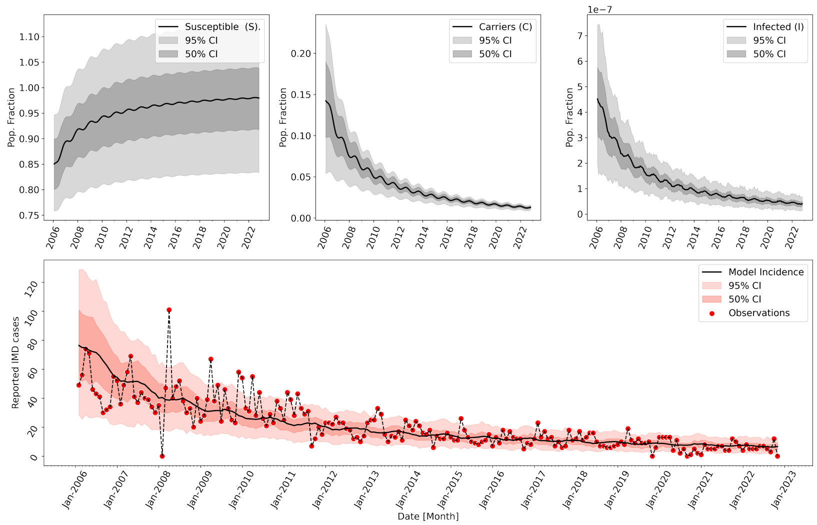


Fig D3: **Model 2. Seasonality in contact rate. IF-EAKF MLE simulation** $\theta$. The upper plots show the time evolution of the state variables from left to right: Susceptibility (S), Prevalence (C), and IMD (I); the lower plot shows incident IMD and reported data as red dots.. Simulation is performed using the MLE of the IF-EAKF.

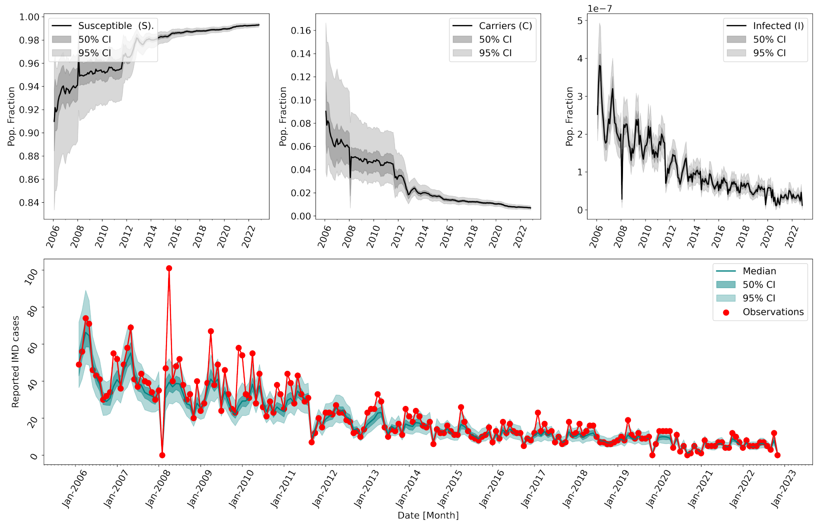


Fig E1: **Model 3. Seasonality in the likelihood of infection. Posterior EAKF state variables**. Upper plots show the posterior estimates of the state variables of an EAKF pass. From left to right we plot Susceptibility (S), Prevalence (C), and IMD (I); the lower plot shows incident IMD and reported data as red dots.

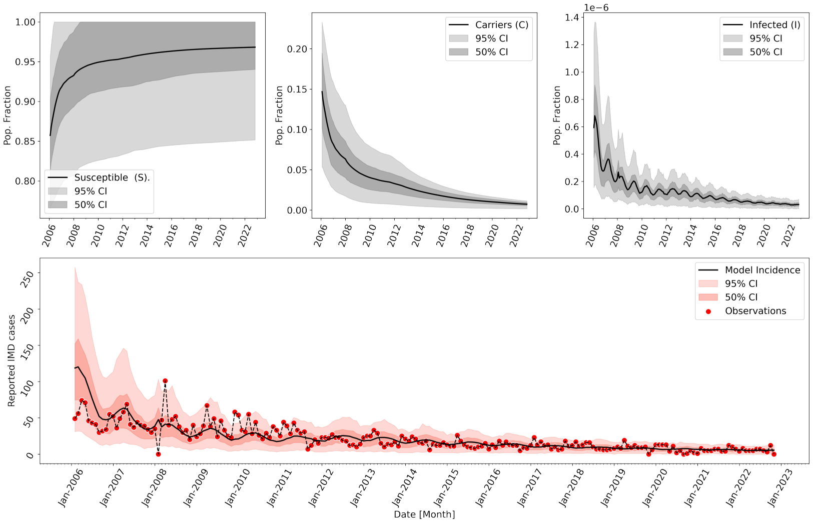


Fig E2: **Model 3. Seasonality in the likelihood of infection. EAKF simulation with posterior parameters.** $\theta$. The upper plots show the time evolution of the state variables from left to right: Susceptibility (S), Prevalence (C), and IMD (I). Simulation is performed using the posterior parameter estimates from a single EAKF pass.

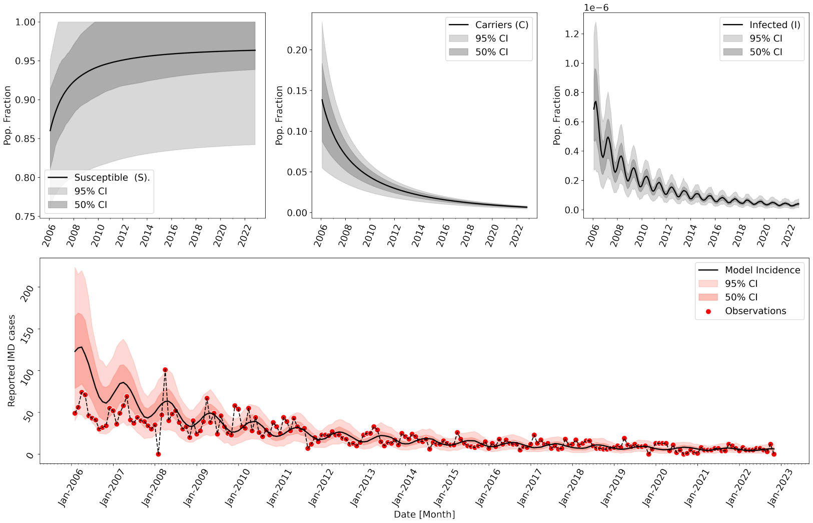


Fig E3: **Model 3. Seasonality in the likelihood of infection. IF-EAKF MLE simulation** $\theta$. The upper plots show the time evolution of the state variables from left to right: Susceptibility (S), Prevalence (C), and IMD (I) and the lower plot shows the incident IMD and the reported data in red dots. Simulation is performed using the MLE of the IF-EAKF.

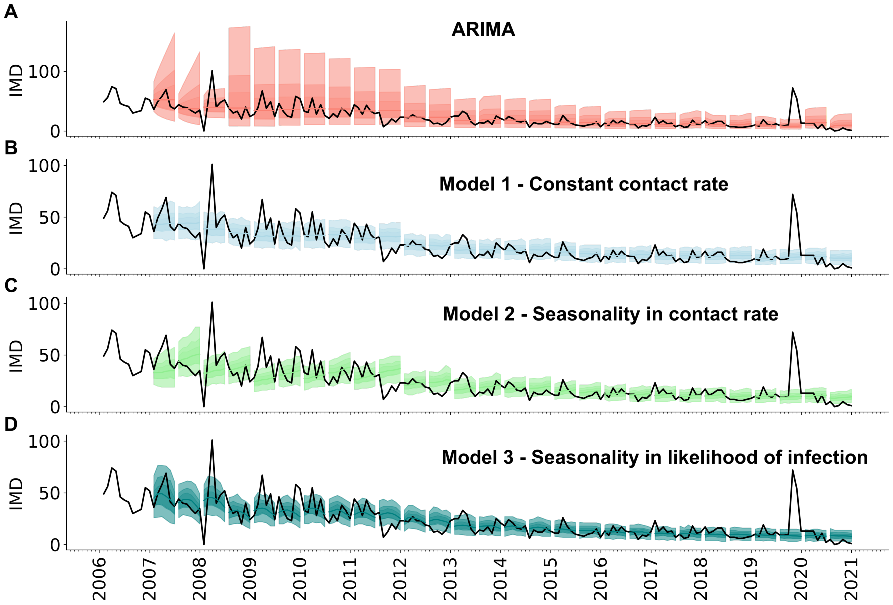


Fig F: **A)** Forecast visualization for the ARIMA model. **B)** Forecast visualization for mechanistic model 1 - constant contact rate. **C)** Forecast visualization for mechanistic model 2 - seasonality in the contact rate. **D)** Forecast visualization for mechanistic model 3 - seasonality in the likelihood of infection. For all subplots, the black lines show IMD incidence, and lighter and darker ribbons show 95% and 50% CI.

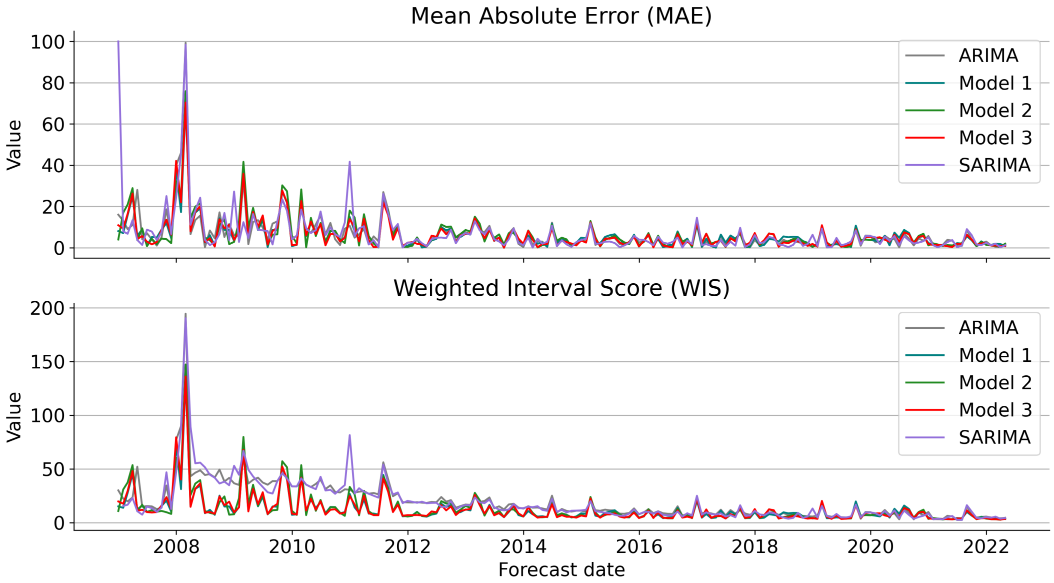


Fig G1: **Performance of individual models at a 1-month forecast horizon.** Upper plot shows the mean absolute error, the lower plot shows WIS.

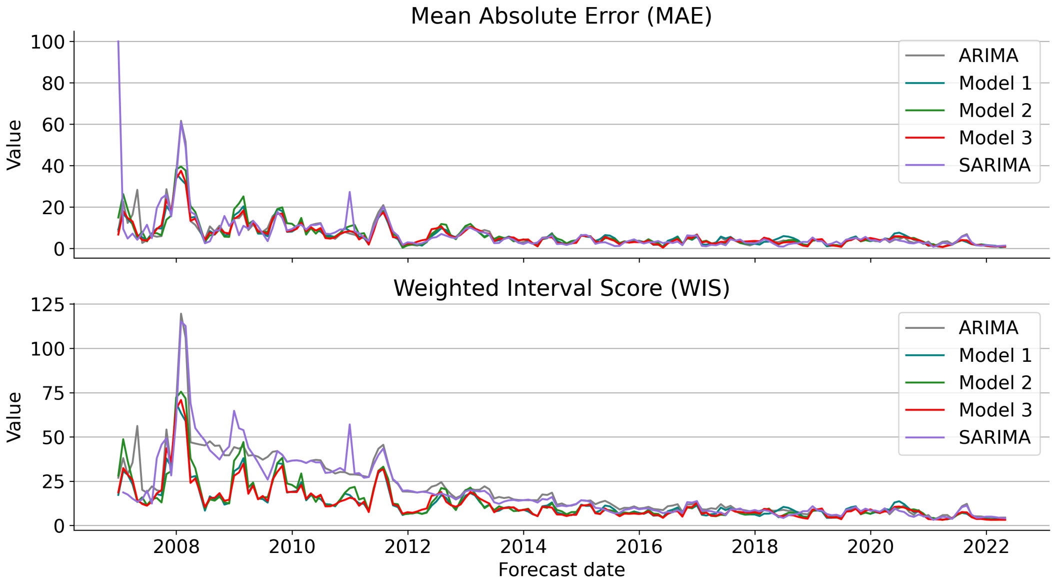


Fig G2: **Performance of individual models at a 3-month forecast horizon.** Upper plot shows the mean absolute error, the lower plot shows WIS.

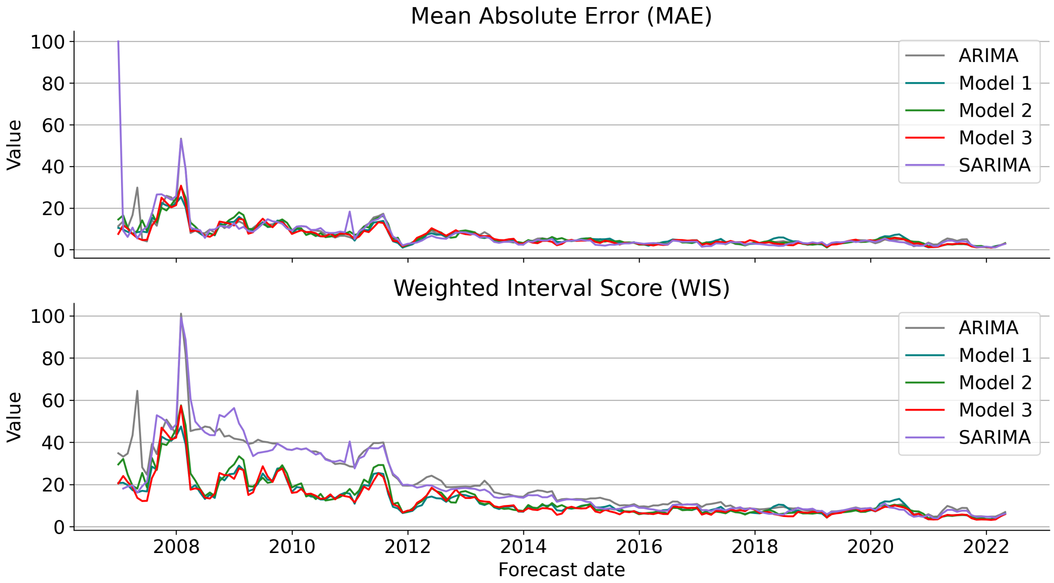


Fig G3: **Performance of individual models at a 6-month forecast horizon.** Upper plot shows the mean absolute error, the lower plot shows WIS.

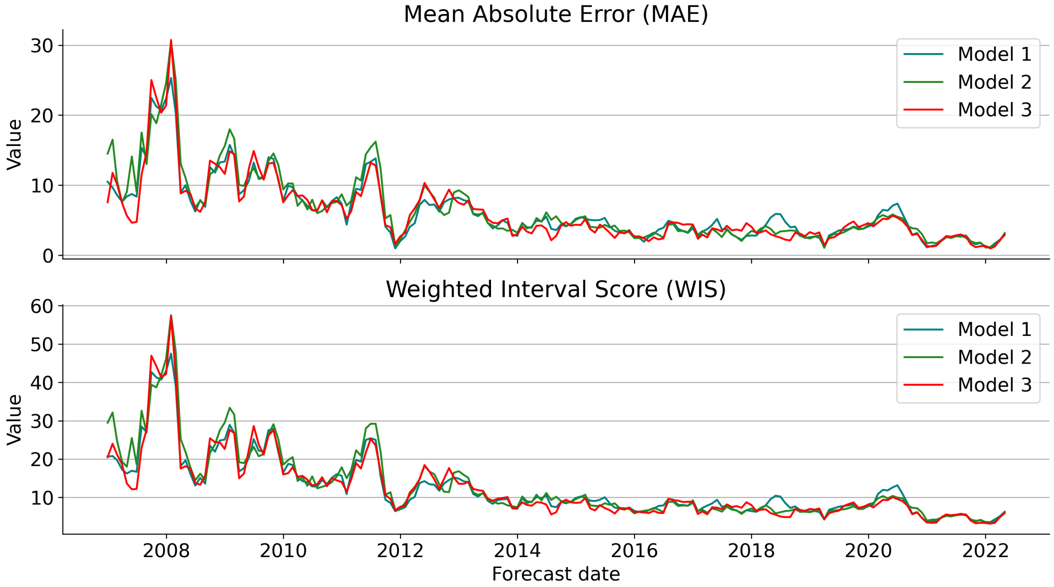


Fig H: **Mechanistic model WIS at a 6-month horizon.** Each line shows the WIS for a specific forecast date. Model labels are indicated in the legend of the plot.

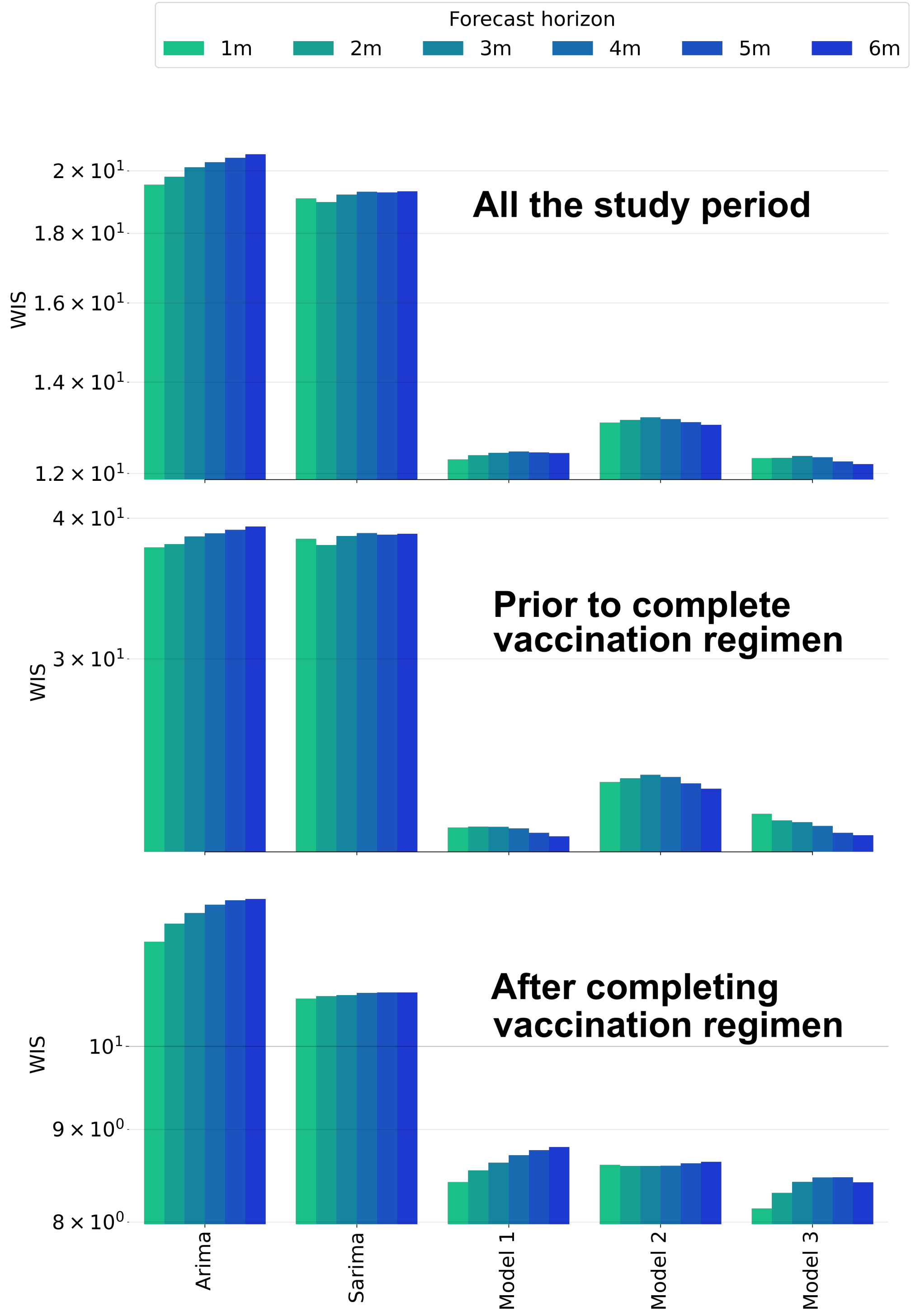


Fig I1: **Mean WIS for individual models.** **A** Mean performance during the study period. **B** Mean performance prior to completion of the vaccine regimen. **C** Mean performance after completing the vaccine regimen.

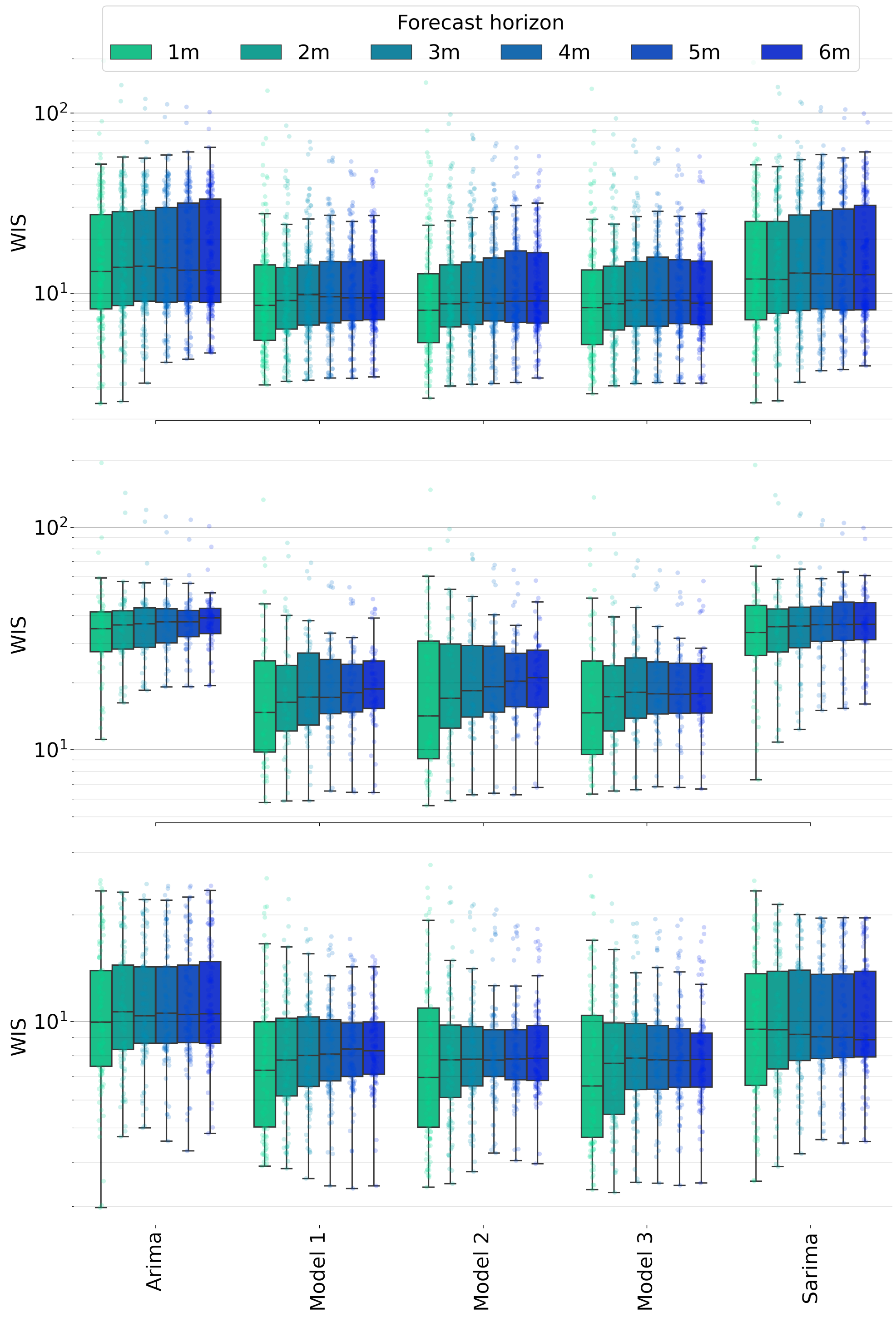


Fig I2: **Boxplot of WIS for individual models. A** Boxplot of WIS during the study period. **B** Boxplot of WIS prior to completion of the vaccine regimen. **C** Boxplot of the WIS after completing the vaccine regimen.

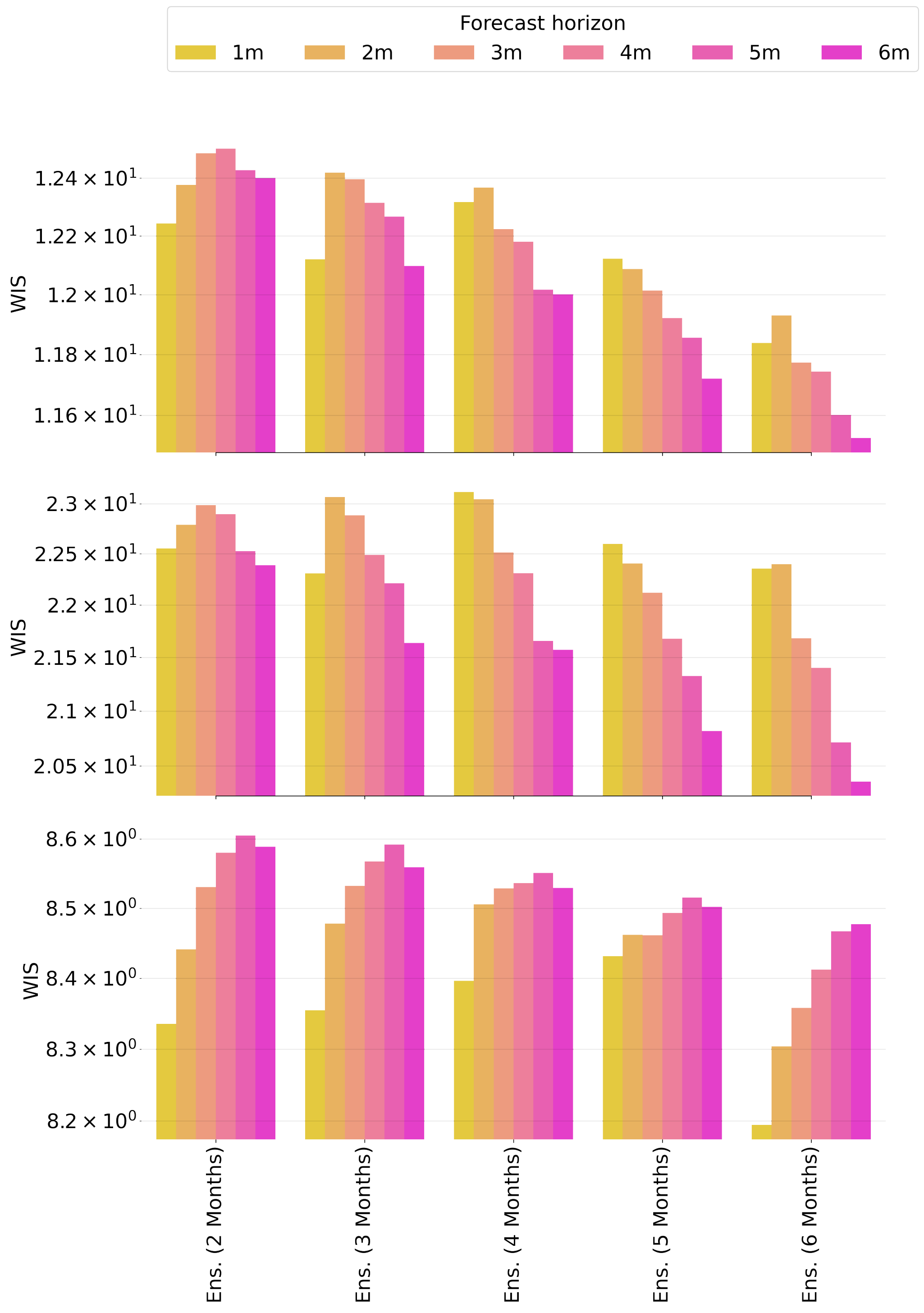


Fig J: **Mean WIS for MME trained with K-months past performance. A)** Mean performance during the study period. **B)** Mean performance prior to completion of the vaccine regimen. **C** Mean performance after completing the vaccine regimen.

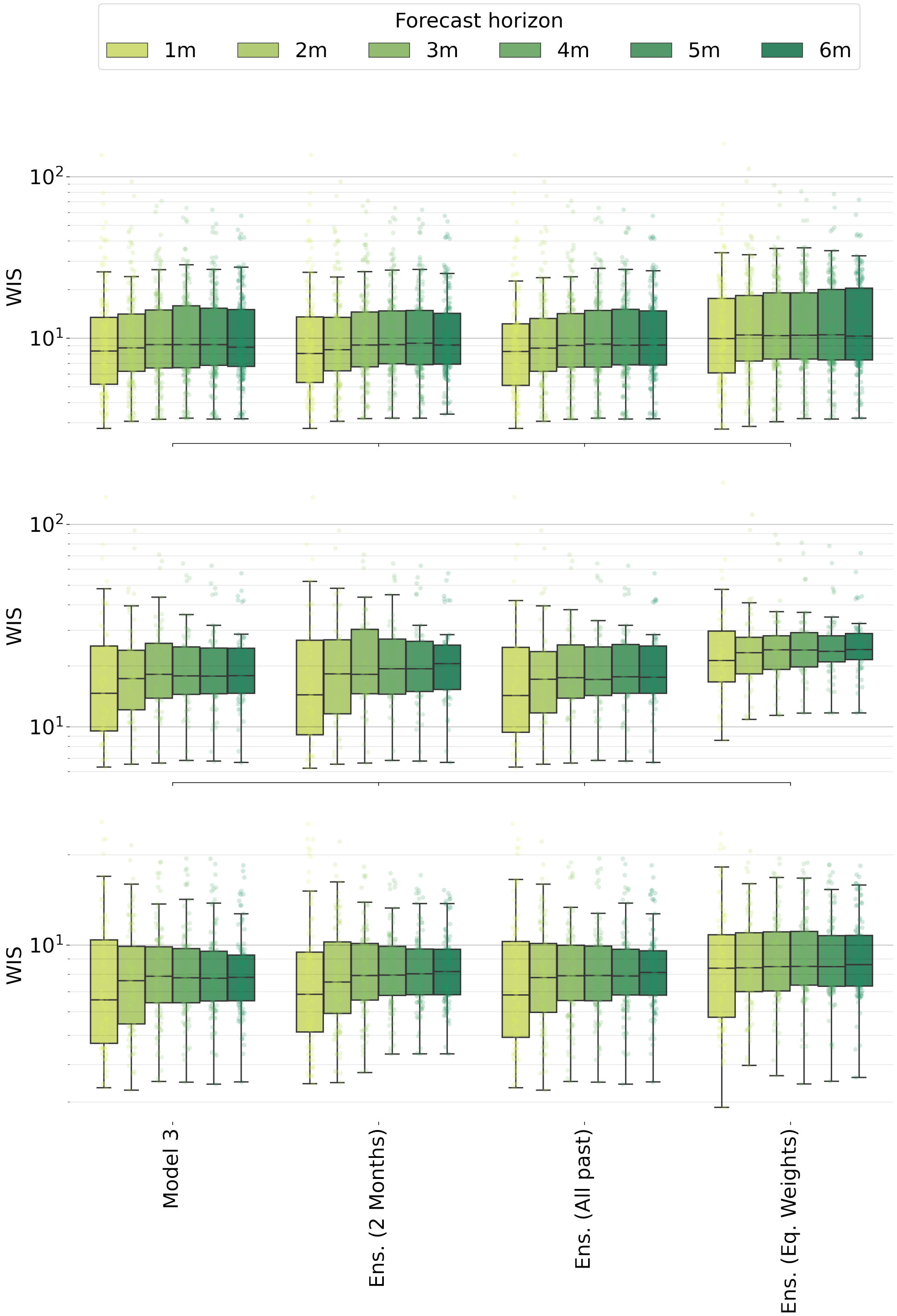


Fig K: **Boxplot of WIS ensembles and best individual model. A** Boxplot of WIS during the study period. **B** Boxplot of WIS prior to completing the vaccine regimen. **C** Boxplot of the WIS after completing the vaccine regimen.

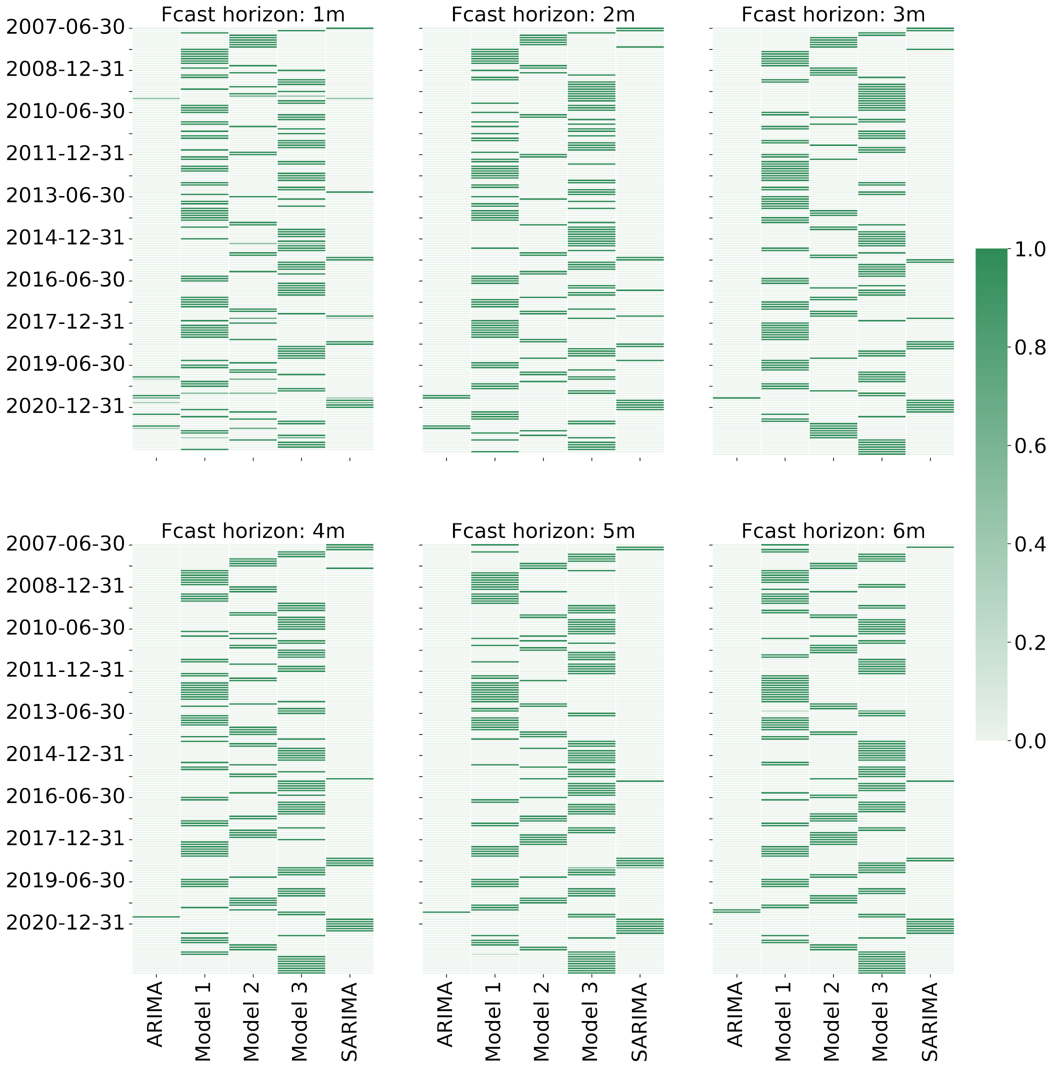


Fig L1: **Weights assigned to each model trained with 2 months of prior performance.** The y-axis shows the forecast data and the x-axis provides the model label. Each subplot shows the results for a different forecast horizon, 1 to 6 months prediction ahead, A to F, respectively.

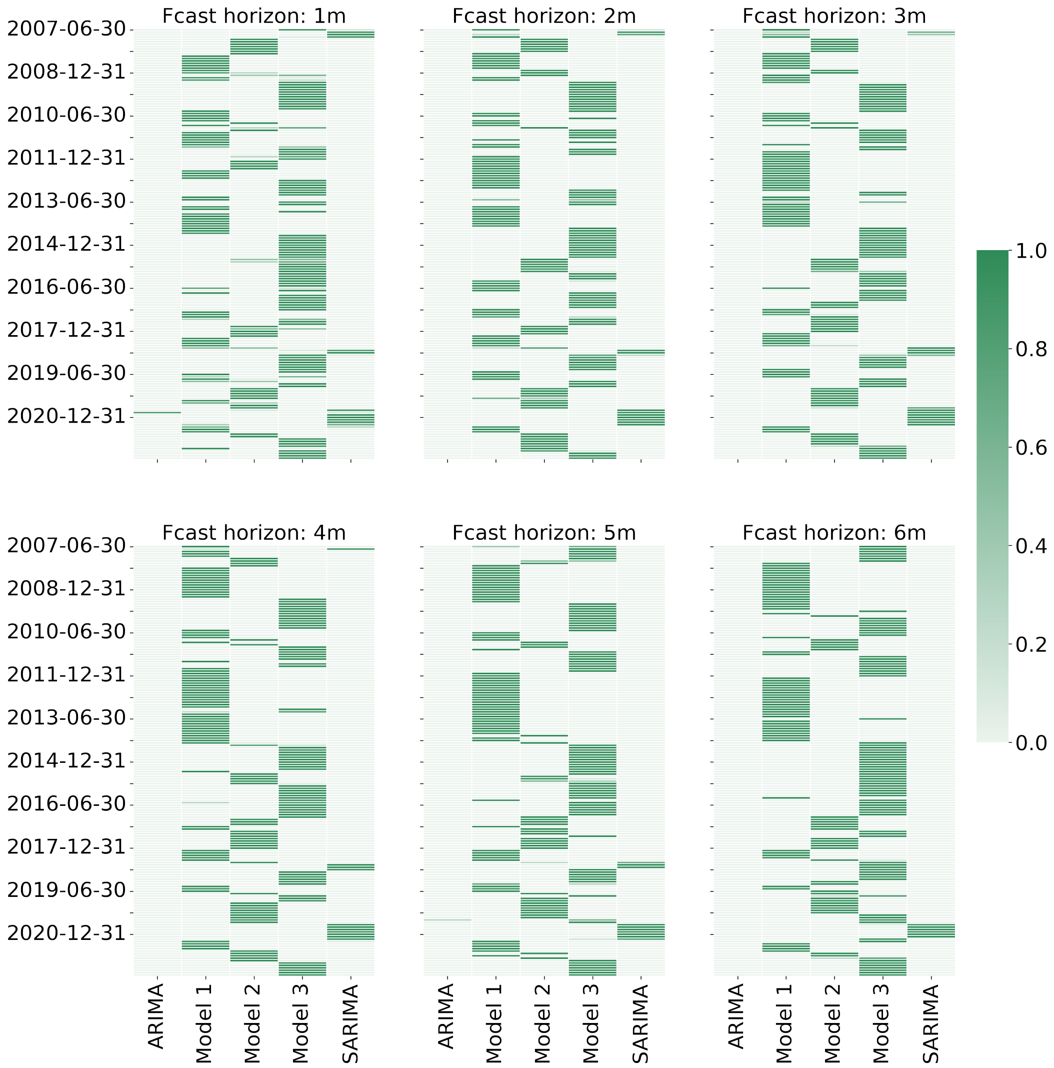


Fig L2: **Weights assigned to each model trained with 6 months of prior performance.** The y-axis shows the forecast date, and the x-axis provides the model label. Each subplot shows the results for a different forecast horizon, 1 to 6 months prediction ahead, A to F, respectively.

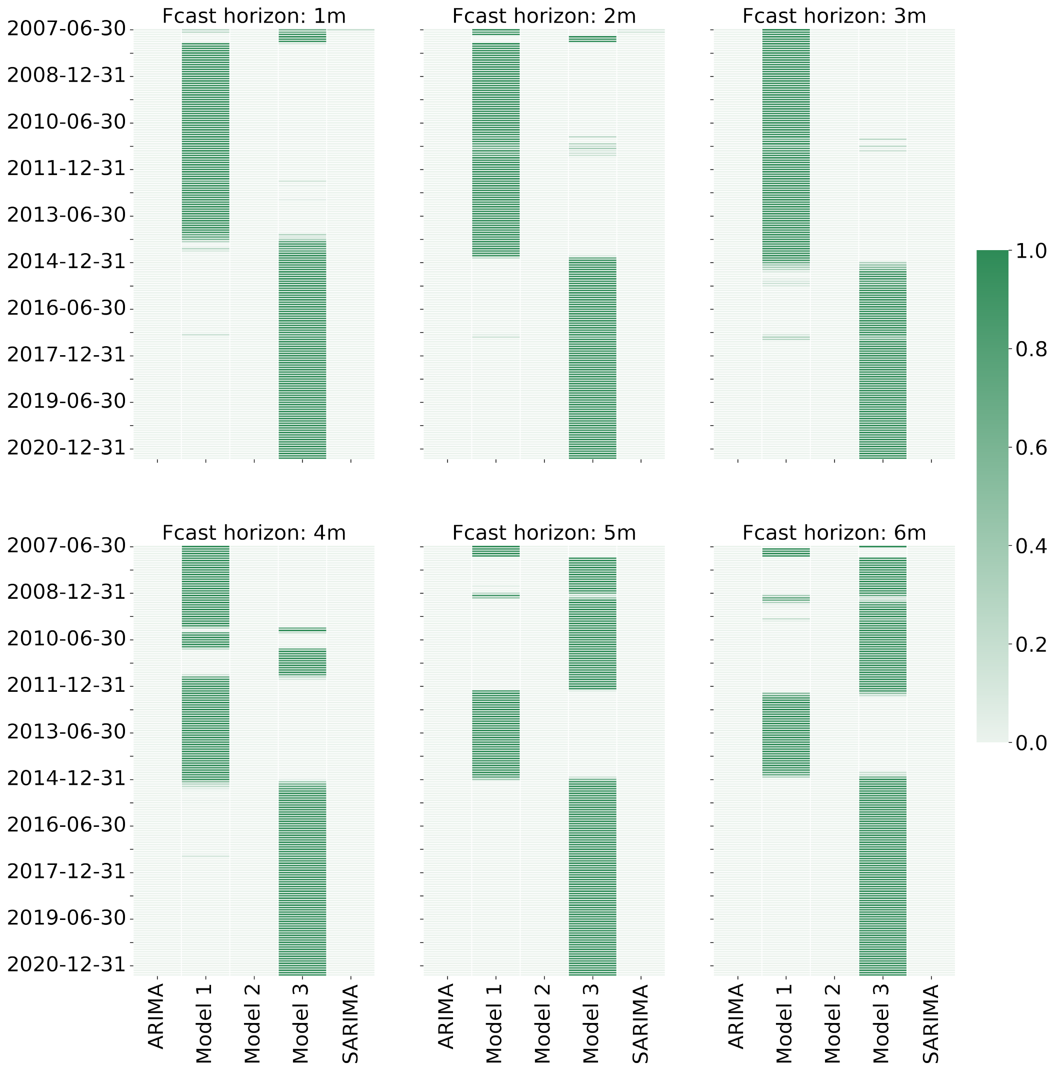


Fig L3: **Weights assigned to each model trained with all past performance.** The y-axis shows the forecast date, and the x-axis provides the model label. Each subplot shows the results for a different forecast horizon, 1 to 6 months prediction ahead, A to F, respectively.

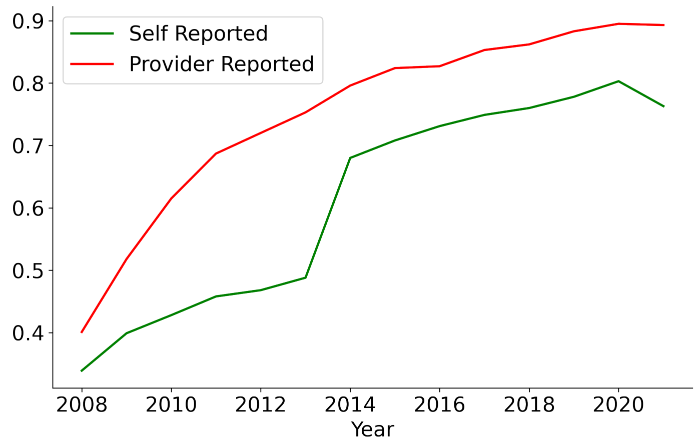


Fig M: Provider and self-reported vaccination in teenagers. Data is from the NIS.

# References


[1] O. Diekmann, J. A. P. Heesterbeek, and J. A. J. Metz. On the definition and the computation of the basic reproduction ratio R0 in models for infectious diseases in heterogeneous populations. *Journal of Mathematical Biology*, 28(4):365–382, June 1990.

[2] Jeffrey L. Anderson. An ensemble adjustment kalman filter for data assimilation. *Monthly Weather Review*, 129(12):2884 – 2903, 2001.

[3] Skipper Seabold and Josef Perktold. statsmodels: Econometric and statistical modeling with python. In *9th Python in Science Conference*, 2010.

[4] Johannes Bracher, Evan L. Ray, Tilmann Gneiting, and Nicholas G. Reich. Evaluating epidemic forecasts in an interval format. *PLOS Computational Biology*, 17(2):1–15, 02 2021.

[5] Nicholas G. Reich, Craig J. McGowan, Teresa K. Yamana, Abhinav Tushar, Evan L. Ray, Dave Osthus, Sasikiran Kandula, Logan C. Brooks, Willow Crawford-Crudell, Graham Casey Gibson, Evan Moore, Rebecca Silva, Matthew Biggerstaff, Michael A. Johansson, Roni Rosenfeld, and Jeffrey Shaman. Accuracy of real-time multi-model ensemble forecasts for seasonal influenza in the U.S. *PLOS Computational Biology*, 15(11):e1007486, November 2019.
