# Supplementary material for: Inference of transmission dynamics and retrospective forecast of invasive meningococcal disease
Source: PLoS Comput Biol. 2023 Oct 27;19(10):e1011564. doi: 10.1371/journal.pcbi.1011564 (PMC10655980; doi:10.1371/journal.pcbi.1011564)
Supplement: Attachment — Submitted filename: Review of Inference of transmission dynamics and retrospective forecast of invasive meningococcal disease.pdf [file pcbi.1011564.s002.pdf]

Review of Inference of transmission dynamics and retrospective forecast of invasive meningococcal disease

Overview

The authors have developed a novel set of models for simulating and forecasting the spread of invasive meningococcal disease (IMD) in the United States. Their study demonstrates the successful integration of mechanistic (process-based) models and Bayesian inference methods in a model-inference system, which effectively captures IMD dynamics over the past 14 years and provides reliable forecasts of future disease outcomes. While similar model-inference systems have been utilized for parameter estimation, evaluation of counterfactual interventions, and forecasting various diseases such as influenza, SARS-CoV-2, West Nile Virus, malaria and dengue, this is the first instance where such methods have been developed specifically for IMD. The analysis serves as a crucial validation and assessment of the IMD model's performance, which will be essential for future investigations on the impact of the SARS-CoV-2 pandemic and vaccinations on IMD incidence. Although the results presented are valuable, there are several areas of weakness that need to be addressed before the manuscript is suitable for publication

Missing demography in process-based models?

The authors have employed a process-based stochastic model to simulate the transmission of invasive meningococcal disease (IMD) in the United States and forecast its incidence. However, a significant assumption underlying these models, which remains unstated, is the exclusion of demographic processes (i.e., birth and deaths) during the study period. Considering that IMD is an endemic disease with a high fatality rate (10-15%), overlooking demographic rates in the model appears to be a substantial omission. It would be beneficial for the authors to provide insights into why birth and death rates have been excluded from their model. Moreover, since the size of the population at risk for IMD in the United States varies annually throughout the 15-year study period, the authors should consider addressing this by discussing the population dynamics and its potential influence on the seasonal transmission of IMD.

Why ARIMA?

It is perplexing that the authors opted to use an autoregressive integrated moving average (ARIMA) model for analyzing data that exhibits a strong seasonal signal. Considering the presence of seasonality in the data, it would have been more appropriate to use the Seasonal Autoregressive Integrated Moving Average (SARIMA) model, which accounts for seasonal variations. Without a proper discussion, the choice of ARIMA appears arbitrary at worst and, at best, a weak comparison. To address this concern, I recommend including a comparison with

SARIMA to validate the performance of the mechanistic seasonal infection model (iii) and the MME."

How robust is the multi-model ensemble method to missing data?

The authors claim "For the MME forecasting system based on the past performance of the individual models, the form using all past predictions for establishing component model weights outperformed forms using only recent predictions" What is the basis for this result? Also do you have a way to estimate the marginal gain/loss in performance from increasing/decreasing the number of past predictions? Answering this question could provide help explain contractions found in previous research.

Unraveling the cause of altered IMD transmission dynamics: how exactly?

The authors note an interesting pattern in IMD cases after 2011 and propose a potential explanation: "Here, we found a marked seasonality of IMD before 2011 with a 1-year period followed by a decreasing seasonal signal after 2011. This qualitative change coincided with changes in vaccine policy and uptake: teenager vaccination for N meningitidis was introduced in 2005 and was extended with an additional booster in 2011. It is possible that vaccination contributed to this shift in seasonality and to the decrease in overall IMD incidence observed in the last 2 decades"

While the vaccination hypothesis is interesting, its support is limited. The effectiveness of a vaccine hinges on the type of protection it provides. Does it prevent susceptible individuals from becoming carriers, or does it primarily reduce the likelihood of transmission within infected individuals or carrier classes? The CDC website indicates that rates of meningococcal disease have declined in the United States since the 1990s, with much of the decline occurring prior to the routine use of MenACWY vaccines. Furthermore, the decline in serogroup B meningococcal disease occurred despite the availability of MenB vaccines only towards the end of 2014.

https://www.cdc.gov/vaccines/vpd/mening/public/index.html#how-well-they-work

Without detailed demographic data, it is important to propose a potential pathway for how vaccination could have led to the observed changes. One possibility is that vaccination reshaped the age distribution of infection, affecting seasonality. Insights from mathematical models on age structure and seasonally transmitted diseases could provide valuable understanding of IMD transmission patterns across vaccine periods. For example, see the following 2019 measles model with seasonally forced transmission rates

https://royalsocietypublishing.org/doi/10.1098/rsif.2019.0151

Minor comments

Introduction

“While meningococcal disease affects all age groups, infections are reported predominantly in infants, young adults, and adults over 85 years old3, and, in temperate regions, cases are predominant in winter and spring months”

What is the distribution of infections? This seems crucial to later claim that MenACWY vaccine (approved in 2005 and 2011) is likely responsible for the significant decline in cases.

Results

Fig 4: The Y-axes have different magnitude. This makes the chart harder to interpret. Suggest rescaling bar charts

“We found that the performance of the MME constructed using all the past performances was better for the entire study period and across all forecast dates and forecast horizons (1 to 6 months). “

Better than what exactly?

We also didn't find any available data on vaccination with the exception of data from NIS teen surveys (Figure S28); however, these survey data are not representative of vaccine hesitancy for the US. Limitations affecting .

There is no Fig S28 in supplementary information.

Discussion

We also reviewed the literature for other possible determinants of transmission and found there have been multiple outbreaks of IMD among men who have sex with men (MSM) in the last 20 decades.

Surely, 20 decades is a bit much.
