# Supplementary material for: Inference of transmission dynamics and retrospective forecast of invasive meningococcal disease
Source: PLoS Comput Biol. 2023 Oct 27;19(10):e1011564. doi: 10.1371/journal.pcbi.1011564 (PMC10655980; doi:10.1371/journal.pcbi.1011564)
Supplement: Attachment — Submitted filename: answer.docx [file pcbi.1011564.s003.docx]

**Review of:** Inference of transmission dynamics and retrospective forecast of invasive meningococcal disease

PLOS computational biology (PCOMPBIOL-D-23-00626)

**Authors: Jaime Cascante-Vega, Marta Galanti, Katharina Schley, Sen Pei, Jeffrey Shaman**

*Answer overview:* We thank the reviewers for their comments. In the resubmission files of the Main text and the Supplementary Information, we have highlighted changes from the previous submission in red to allow the reviewers and editor(s) ease of tracking. The principal changes in the Main text include i) a description of the birth and death dynamics in the model; and ii) a description of the SARIMA model. All other changes are indicated in the answers below and highlighted in red in the main text.

We have updated all figures, in both the Main text and SI, as the MMEs now include the SARIMA, and birth and death dynamics are now included.

**Reviewer 1.**
*Overview*
The authors have developed a novel set of models for simulating and forecasting the spread of invasive meningococcal disease (IMD) in the United States. Their study demonstrates the successful integration of mechanistic (process-based) models and Bayesian inference methods in a model-inference system, which effectively captures IMD dynamics over the past 14 years and provides reliable forecasts of future disease outcomes. While similar model-inference systems have been utilized for parameter estimation, evaluation of counterfactual interventions, and forecasting various diseases such as influenza, SARS-CoV-2, West Nile Virus, malaria and dengue, this is the first instance where such methods have been developed specifically for IMD. The analysis serves as a crucial validation and assessment of the IMD model's performance, which will be essential for future investigations on the impact of the SARS-CoV-2 pandemic and vaccinations on IMD incidence. Although the results presented are valuable, there are several areas of weakness that need to be addressed before the manuscript is suitable for publication.

*Missing demography in process-based models?*

The authors have employed a process-based stochastic model to simulate the transmission of invasive meningococcal disease (IMD) in the United States and forecast its incidence. However, a significant assumption underlying these models, which remains unstated, is the exclusion of demographic processes (i.e., birth and deaths) during the study period. Considering that IMD is an endemic disease with a high fatality rate (10-15%), overlooking demographic rates in the model appears to be a substantial omission. It would be beneficial for the authors to provide insights into why birth and death rates have been excluded from their model. Moreover, since the size of the population at risk for IMD in the United States varies annually throughout the 15-year study period, the authors should consider addressing this by discussing the population dynamics and its potential influence on the seasonal transmission of IMD.

*Answer:* We thank the reviewer for suggesting the inclusion of population dynamics. We now include birth and death rates in the model. We assume all individuals are born susceptible to acquiring the bacterium. We, therefore, model births as $b\sim Binom(N, 1-exp(-\mu))$, and deaths $d_{x}$ in each compartment $X$ as $d_{x}\sim Binom(X, 1-exp(-\mu))$, where $\mu$ is the birth/death rate. We disregarded additional mortality due to IMD due to its negligible impact on model dynamics. The new model version produced highly similar inferences as presented with the previous models. We present new simulations with time-varying estimated parameters from an EAKF, the posterior estimate of the state space, and simulation with point parameters estimates using an IF-EAFK (figures included in separate pdf file). We find, for each individual dynamical model including births and deaths, simulation with posterior parameters estimates using an EAKF and with point parameters estimates using an IF-EAKF results in incidence estimates that conserve the trend in the data (except for model 2, for which simulations with time varying posterior parameters estimates using the EAKF substantially overestimated IMD incidence). Lastly, we compare forecast accuracy, using WIS, for models with and without population dynamics (Figure R1). We found that both types of models produce similar results. However, as the forecast horizon increases from 1m to 6m the models including population dynamics performed slightly better (lower dashed red lines compared to solid black lines).

Figure R1. Weighted interval score (WIS) of models with birth and deaths (BD in legend, red lines) and without BD dynamics (black lines). Forecast horizon increases from upper to lower rows; we only present 2, 4, and 6 month leads. In each column, we present one of the dynamical models introduced in the main text: model without seasonality (model 1), model with seasonal transmission rate (model 2), and model with seasonal likelihood of infection (model 3).

*Why ARIMA?*

It is perplexing that the authors opted to use an autoregressive integrated moving average (ARIMA) model for analyzing data that exhibits a strong seasonal signal. Considering the presence of seasonality in the data, it would have been more appropriate to use the Seasonal Autoregressive Integrated Moving Average (SARIMA) model, which accounts for seasonal variations. Without a proper discussion, the choice of ARIMA appears arbitrary at worst and, at best, a weak comparison. To address this concern, I recommend including a comparison with SARIMA to validate the performance of the mechanistic seasonal infection model (iii) and the MME."

*Answer:* Thank you for this comment. We now include comparisons with SARIMA. We present the performance of all the individual dynamical and statistical models across the 3 data periods introduced in the main text: before the complete vaccination regime (2011), after it, and during the whole study period (figures below). We produce the MME using the past performance (See **Multi-model ensemble of forecasting models and evaluation** in section **Materials and Methods** of the Main text) of the 3 individual dynamical models and the 2 statistical models (ARIMA and SARIMA). We evaluate the performance with a proper scoring rule for probabilistic forecasts: the WIS. We find, as with the previous results, the dynamical models outcompete the statistical ones. The broad uncertainty of the forecast for both the ARIMA and the SARIMA is penalized by the probabilistic scoring rule. In Figure R2, we present (1) the mean WIS of all 5 individual models across the data periods and (2) the distribution of the WIS of the models across the data periods. Our finding that mechanistic models perform better across different forecast horizons remains after the addition of the SARIMA model. We find that the SARIMA had a better performance than the ARIMA.

In the new main text, we have included a brief description of the SARIMA model and have updated all the figures and tables. We also have edited the figures in the Supplementary Information and the statistical analyses used to assess significant differences between the models.

Figure R2. Mean weighted interval score (WIS) for 3 different data periods. **Upper:** During the entire study period. **Middle:** Before completing the vaccination regimen. **Lower:** After completing the vaccination regimen. The forecast horizon is color-coded, and the models' names are indicated on the x-axis and shared across plots.

*How robust is the multi-model ensemble method to missing data?*

The authors claim “For the MME forecasting system based on the past performance of the individual models, the form using all past predictions for establishing component model weights outperformed forms using only recent predictions” What is the basis for this result? Also do you have a way to estimate the marginal gain/loss in performance from increasing/decreasing the number of past predictions? Answering this question could provide help explain contractions found in previous research.

*Answer:* We thank the reviewer for this suggestion. In the previous submission, we included Supplementary Figures (Fig 21) to justify our claim. SI Figure 21 shows the mean WIS of the MME built with the past performance from 1m to 6m. We presented this across the 3 data periods (splits) introduced in the main text, marked by the completion of the vaccination regimen. We now have estimated the marginal loss in performance as the number of past predictions used to build the MME decreases. The approach is a simple linear regression where the predicted variable is the mean WIS, and the predictor is the number of months used to train the MME. In figure R3, we present these findings. We fit different linear models considering the 3 data splits introduced and the forecast horizons used to evaluate the models’ performance. We find that for combinations of the 3 different periods and forecast horizons, a model with a negative slope accurately predicts the gain in performance (decrease in WIS). We also find that the magnitude of the slope increased as the forecast horizon increased, which translates in a more significant improvement as the MME forecast is interrogated with more data. While a negative slope explains the change in performance for all the 3 data periods considered; the improvement in MME performance is more pronounced (higher magnitude of the slope) for the first data period (before the completion of vaccination). While our approach reveals that increasing information (i.e. the number of months used to build the MME) increases performance, it does not illuminate possible mechanisms underlying this relationship, which could be leveraged in future research.

Figure R3. Mean weighted interval score (WIS) as the number of past months used to train the MME is increased. The data split is indicated in each row and the forecast horizon is indicated in each column. The red dot is the mean WIS of the model trained with all past performance, located for simplicity at x=7. Regressions were performed with all dots (black continuous line) and with purple dots (dashed purple line) to exclude the performance of the MME trained with all past performance.

*Unraveling the cause of altered IMD transmission dynamics: how exactly?*

The authors note an interesting pattern in IMD cases after 2011 and propose a potential explanation: “Here, we found a marked seasonality of IMD before 2011 with a 1-year period followed by a decreasing seasonal signal after 2011. This qualitative change coincided with changes in vaccine policy and uptake: teenager vaccination for N meningitidis was introduced in 2005 and was extended with an additional booster in 2011. It is possible that vaccination contributed to this shift in seasonality and to the decrease in overall IMD incidence observed in the last 2 decades”

While this potential explanation is interesting, its support based on our dataset is limited. The effectiveness of a vaccine hinges on the type of protection it provides. Does it prevent susceptible individuals from becoming carriers, or does it primarily reduce the likelihood of transmission within infected individuals or carrier classes? The CDC website indicates that rates of meningococcal disease have declined in the United States since the 1990s, with much of the decline occurring prior to the routine use of MenACWY vaccines. Furthermore, the decline in serogroup B meningococcal disease has begun before? the availability of MenB vaccines towards the end of 2014.

https://www.cdc.gov/vaccines/vpd/mening/public/index.html#how-well-they-work

Without detailed demographic data, it is important to propose a potential pathway for how vaccination could have led to the observed changes. One possibility is that vaccination reshaped the age distribution of infection, affecting seasonality. Insights from mathematical models on age structure and seasonally transmitted diseases could provide valuable understanding of IMD transmission patterns across vaccine periods. For example, see the following 2019 measles model with seasonally forced transmission rates

https://royalsocietypublishing.org/doi/10.1098/rsif.2019.0151

*Answer:* Unfortunately, the data available are not stratified by age or age group before 2015. The yearly age distribution of infections starting in 2015 can be found here: ^1–6^. We therefore can only speculate and have modified our statement to the one shown below, while providing a few citations. The citations include the model insights the reviewer provides as well as empirical evidence of the effect on carriage and IMD of the men B and ACWY conjugate vaccines.

"Here, we found a marked seasonality of IMD before 2011 with a 1-year period followed by a decreasing seasonal signal after 2011. This qualitative change coincided with changes in vaccine policy and uptake: teenager vaccination for N meningitidis was introduced in 2005 and was extended with an additional booster in 2011. … the ACWY conjugate vaccine reduces carriage^7^, impacting transmission dynamics via reducing the force of infection. However, it is also possible that the observed change in seasonality is explained by changes in the age distribution of infection^8^."

***Minor comments***

*Introduction*

“While meningococcal disease affects all age groups, infections are reported predominantly in infants, young adults, and adults over 85 years old3, and, in temperate regions, cases are predominant in winter and spring months”

What is the distribution of infections? This seems crucial to later claim that MenACWY vaccine (approved in 2005 and 2011) is likely responsible for the significant decline in cases.

*Answer:* Please see the previous point*.*

*Results*

Fig 4: The Y-axes have different magnitude. This makes the chart harder to interpret. Suggest rescaling bar charts

*Answer:* Thank you for the suggestion. We presented each data split with its magnitude because IMD incidence numbers were considerably higher before versus after 2011 - and we aimed to compare models and MMEs, not performance between data splits/time periods. Figure R4 shows below the y-axis with the same limits for each data split. As you can see, the visual comparison between models is degraded, therefore we have maintained the original figure in the manuscript.

Figure R4. **Performance of model and ensemble forecasts.** Each bar shows the mean performance for the indicated split of the data; forecast horizon is color-coded and indicated in the legend**. A)** The entire study period **B)** Pre 2011. **C)** Post 2011.

“We found that the performance of the MME constructed using all the past performances was better for the entire study period and across all forecast dates and forecast horizons (1 to 6 months). “

Better than what exactly?

*Answer:* We have clarified in the manuscript: "We found that MME constructed using all past performances performed better across all periods and horizons than MME constructed using only more recent performance.”

*Missing SI figure*

We also didn't find any available data on vaccination with the exception of data from NIS teen surveys (Figure S28); however, these survey data are not representative of vaccine hesitancy for the US. Limitations affecting.

There is no Fig S28 in the supplementary information.

*Answer:* We thank the reviewer for noticing this. We now included the figure as Fig S26 in the supplementary materials.

*Discussion*

We also reviewed the literature for other possible determinants of transmission and found there have been multiple outbreaks of IMD among men who have sex with men (MSM) in the last 20 decades.

Surely, 20 decades is a bit much.

*Answer:* We apologize for the slip; we have changed ‘’decades’’ to ‘’years’’. The last outbreak in the MSM community was at the beginning of this year (2023) in Florida. We thought it was important to suggest possible super-spreading events that, in the case of IMD could be driven by a specific community. Super-spreading events violate the assumption of perfect population mixing as represented in the compartmental models and could potentially explain poor fit to the unusual peaks in the data, as in 2008.

**Reviewer 2.**

*Overview*

This is a well written manuscript, in which the authors set out to model invasive meningococcal disease in the US, using a simple approach based mainly on seasonality of risk of invasive disease and general carriage trends around vaccine programs.
I’m not an expert of modeling techniques, in particular not Bayesian methods, so cannot comment on the methodology used.

*Utility of wavelet analysis*

Overall, the authors conclude that a wavelet analysis with seasonality can reasonably well predict overall evolution of IMD case number in the US. However, the utility of such approach is less clear: how does this guide vaccine recommendation, by serogroup and by age group?

*Answer:* While the ultimate goal of modeling is to support disease response and to guide vaccine recommendations, the goal of the LWPS was not to provide such insights. Instead, as it has been used for many other infectious disease systems, Fourier decomposition methods or LWPS have been used to understand seasonal patterns in time^9,10^. We also used the LWPS to obtain the periodicity to inform the seasonal mechanistic dynamical models.

Additional stratification of the time series of IMD incidence by age and serogroup could directly be input to the LWPS to understand seasonal patterns in those time series. The CDC Wonder app provides stratification by serogroup beginning in 2020. In **Data description** in **Materials and Methods** we wrote: ‘*Classification of cases by serogroup (ACWY and B) only began in 2020 and was not used in this study; rather, incidence includes all serogroups*’. Due to this paucity of data, we would not be able to leverage the model/inference system to forecast IMD incidence with serogroup stratification.

To understand and quantify the relationship between the time series of vaccination and IMD incidence, cross-wavelet mean fields (CWMF) might be used. CWMF has been used to quantify the relationship between temperature and dengue incidence in space and time^11^. Further split of the IMD incidence data by age and vaccination data, both unavailable to our knowledge, could be used to quantify synchrony across space (age groups) and to quantify the impact of vaccination on IMD incidence. We appreciate the question as it allows us to think about future avenues of investigation. However, including this, particularly given data limitations, is beyond the scope of the current work. We now discuss these issues in the revised manuscript in the 5th paragraph of the discussion: "Stratification of the IMD incidence dataset by age, serogroup and vaccination status could support testing these hypotheses through more detailed IMD modeling"

*Performance on 'outliers'*

Extreme incidences like in 2008-2010 are poorly modeled. This may be explained by one major limitation of the work (recognized by the authors): the fact that the trends in influenza incidences are not included. On the same vein, it appears simplistic not to include strain evaluations or serogroup distributions in the modeling effort.

*Answer:*

Previous research (see the 5th paragraph of the discussion) used purely statistical methods and showed correlations between influenza-like illness and hospitalizations of IMD^12^. This led us to hypothesize that prior exposure to influenza could increase the likelihood of infection, given colonization. This reasoning may also explain why a time-varying likelihood of infection in our model better explains the data. This has been shown for *Streptococcus pneumoniae* using model-inference methods^13^. However, spatially finer IMD data would be needed to confirm this. Future research, perhaps leveraging a more complete IMD dataset, could shed some light on the link between influenza and IMD. Another possible source of the unusual peak observed in 2008 could be super-spreading events, which we also discuss - driven by outbreaks within the men who have sex with men (MSM) community^1^.

We did not add strain evaluations or serogroup distributions due to the limited availability of such data, as indicated previously. However, we now mention these issues in the revised manuscript in the 5th paragraph of the discussion: "Lastly, future research, perhaps leveraging a more complete IMD dataset resolved at finer spatial scales, could shed some light on the link between influenza and IMD."

*Climate change*

I would appreciate a discussion of predictions taking into account climate change in the US: how would this be captured by the model?
*Answer:* Thanks for this comment; it is important to consider the effects of climate change. While our work suggests climate plays a possible role supporting the seasonal likelihood of infection, other factors might be in play, as discussed (see the 3rd and 4th paragraphs of the **Discussion** section). If climate is the principal driver of seasonality for the IMD system, it would be crucial to account for climate change in the prediction system. However, the mechanism behind the observed seasonality remains to be elucidated. A recent systematic literature review linking climatic hazards with infectious diseases^14^ highlights possible climate factors mechanism explaining IMD incidence. The work shows *droughts*, *storms,* and *warming* as climatic effects facilitating IMD and *Neisseria meningitidis* spread, for an interactive webpage overview the reviewer can access here: ^15^. This is an important future area of research. We have added text to the Discussion further underscoring these issues: "If climate is shown to be a driver of the seasonality observed, the effects of a changing climate would need to be incorporated into IMD models".

*African meningitis belt*

The authors argue for some assumptions with reference to evidence from the African meningitis belt. However, the role of the climate in the carriage and invasive disease is not transposable to the situation of the US, and I recommend being less affirmative in this perspective.

*Answer:* Thank you for pointing this out. We did not intend to translate findings from the African meningitis belt to the US, rather to highlight a possible mechanism behind seasonality in infection broadly. We have clarified this in the manuscript discussion: "Despite the different conditions in the US, evidence from the meningitis Belt supports investigation of a potential effect of climate on disease mechanisms".

*Predicted prevalence matches empirical observations?*

It is not clear in how far the predicted carriage prevalences are compatible with observed data – this would be important to address.

*Answer:* Thank you for noting that this statement is not clear. We selected the initial conditions of the prevalence sampled uniformly between 5% and 30% following cross-sectional studies in the literature^16^. We further: “*derived a prior range of parameters and variables by imposing at equilibrium that the basic reproductive number and the carriage prevalence is around 20%, we study the sensitivity of the restriction to different decolonization periods (See Supplementary Information Figures SI2-SI4)”.* However, we are unaware of any studies systematically reporting carriage data across time. Consequently, we could not validate the estimated prevalence in time.

*COVID19*

From the manuscript, it remains unclear how the incidence of IMD changed across the Covid-19 pandemic, during and following the restrictions. This should be specifically addressed, globally in the US and regarding local outbreaks.

*Answer:* We agree that understanding the effect of non-pharmaceutical interventions (NPIs) on the carriage prevalence of *N. meningitidis* and on IMD incidence would be very interesting.

We now have extended our analyses through the end of 2022 and show that the model-inference system does not estimate changes in prevalence or likelihood of infection at the national level. However, as the review suggests, a deeper understanding of local outbreaks contextualized with knowledge of city and state-level NPIs would be needed. This is not one of the objectives of our work, but we agree that it would be interesting to expand this idea in future research. Some modeling studies in the UK, accounting for the effects of decreased vaccination during the pandemic, suggest long term effects of NPIs on carriage prevalence^17^. However, further modeling, validated with recent, local data are needed to better assess the effect of the COVID-19 pandemic on IMD. We have clarified this in the manuscript discussion.

*Coverage estimates of meningococcal vaccines*

Finally, please provide coverage estimates for the recommended and not systematically recommended meningococcal vaccines in the US.

*Answer:* In Supplementary Information Fig S26 we provided coverage estimates of Meningococcal vaccination for teenagers aged 13-17 years old in US. This information is available via the yearly NIS teens survey and is available from 2008.

*Supplementary material artifact*
In the supplementary material, some models show an IMD peak in 2019 – is this an artefact? Please comment on this observation in the discussion.

*Answer:* The spike derived from an artifact in the 2021 CDC wonder app dataset, that appears corrected in the 2022 data release. We have now corrected the Figures.

**References**

1.	Center for Disease Control and Prevention. Revised Recommendations of the Advisory Committee on Immunization Practices to Vaccinate All Persons Aged 11--18 Years with Meningococcal Conjugate Vaccine. https://www.cdc.gov/mmwr/preview/mmwrhtml/mm5631a3.htm.

2.	MacNeil, J. R., Rubin, L. G., Patton, M., Ortega-Sanchez, I. R. & Martin, S. W. Recommendations for Use of Meningococcal Conjugate Vaccines in HIV-Infected Persons — Advisory Committee on Immunization Practices, 2018. *MMWR Morb. Mortal. Wkly. Rep.* **65**, 1189–1194 (2018).

3.	MacNeil, J. R., Rubin, L. G., Patton, M., Ortega-Sanchez, I. R. & Martin, S. W. Recommendations for Use of Meningococcal Conjugate Vaccines in HIV-Infected Persons — Advisory Committee on Immunization Practices, 2017. *MMWR Morb. Mortal. Wkly. Rep.* **65**, 1189–1194 (2017).

4.	MacNeil, J. R., Rubin, L. G., Patton, M., Ortega-Sanchez, I. R. & Martin, S. W. Recommendations for Use of Meningococcal Conjugate Vaccines in HIV-Infected Persons — Advisory Committee on Immunization Practices, 2015. *MMWR Morb. Mortal. Wkly. Rep.* **65**, 1189–1194 (2015).

5.	MacNeil, J. R., Rubin, L. G., Patton, M., Ortega-Sanchez, I. R. & Martin, S. W. Recommendations for Use of Meningococcal Conjugate Vaccines in HIV-Infected Persons — Advisory Committee on Immunization Practices, 2019. *MMWR Morb. Mortal. Wkly. Rep.* **65**, 1189–1194 (2019).

6.	MacNeil, J. R., Rubin, L. G., Patton, M., Ortega-Sanchez, I. R. & Martin, S. W. Recommendations for Use of Meningococcal Conjugate Vaccines in HIV-Infected Persons — Advisory Committee on Immunization Practices, 2021. *MMWR Morb. Mortal. Wkly. Rep.* **65**, 1189–1194 (2021).

7.	Carr, J. P. *et al.* Impact of meningococcal ACWY conjugate vaccines on pharyngeal carriage in adolescents: evidence for herd protection from the UK MenACWY programme. *Clinical Microbiology and Infection* **28**, 1649.e1-1649.e8 (2022).

8.	Magpantay, F. M. G., King, A. A. & Rohani, P. Age-structure and transient dynamics in epidemiological systems. *J. R. Soc. Interface.* **16**, 20190151 (2019).

9.	Grenfell, B. T., Bjørnstad, O. N. & Kappey, J. Travelling waves and spatial hierarchies in measles epidemics. *Nature* **414**, 716–723 (2001).

10.	Paireau, J., Chen, A., Broutin, H., Grenfell, B. & Basta, N. E. Seasonal dynamics of bacterial meningitis: a time-series analysis. *The Lancet Global Health* **4**, e370–e377 (2016).

11.	García-Carreras, B. *et al.* Periodic synchronisation of dengue epidemics in Thailand over the last 5 decades driven by temperature and immunity. *PLoS Biol* **20**, e3001160 (2022).

12.	Jacobs, J. H. *et al.* The Association of Meningococcal Disease with Influenza in the United States, 1989–2009. *PLoS ONE* **9**, e107486 (2014).

13.	Shrestha, S. *et al.* Identifying the Interaction Between Influenza and Pneumococcal Pneumonia Using Incidence Data. *Sci. Transl. Med.* **5**, (2013).

14.	Mora, C. *et al.* Over half of known human pathogenic diseases can be aggravated by climate change. *Nat. Clim. Chang.* **12**, 869–875 (2022).

15.	Mora C, McKenzie T, Gaw IM, Dean JM, von Hammerstein H, Knudson TA, Setter RO, Smith CZ, Webster KM, Patz JA, Franklin EC. Traceable evidence of the impacts of climate change on pathogenic human diseases: Over half of known human pathogenic diseases can be aggravated by climate change. https://camilo-mora.github.io/Diseases/?type=,5&id=,146&impact=Negative (2022).

16.	Christensen, H., May, M., Bowen, L., Hickman, M. & Trotter, C. L. Meningococcal carriage by age: a systematic review and meta-analysis. *The Lancet Infectious Diseases* **10**, 853–861 (2010).

17.	Hadley, L., Karachaliou Prasinou, A., Christensen, H., Ramsay, M. & Trotter, C. Modelling the impact of COVID-19 and routine MenACWY vaccination on meningococcal carriage and disease in the UK. *Epidemiol. Infect.* **151**, e98 (2023).
